# Supplementary material for: Identification of State-Specific Proteomic and Transcriptomic Signatures of Microglia-Derived Extracellular Vesicles
Source: Mol Cell Proteomics. 2023 Nov 11;22(12):100678. doi: 10.1016/j.mcpro.2023.100678 (PMC10755493; doi:10.1016/j.mcpro.2023.100678)

Raw Western blot:
BV2 cells
EV lysates and cell lysates
Probed with CD9 (22kda)

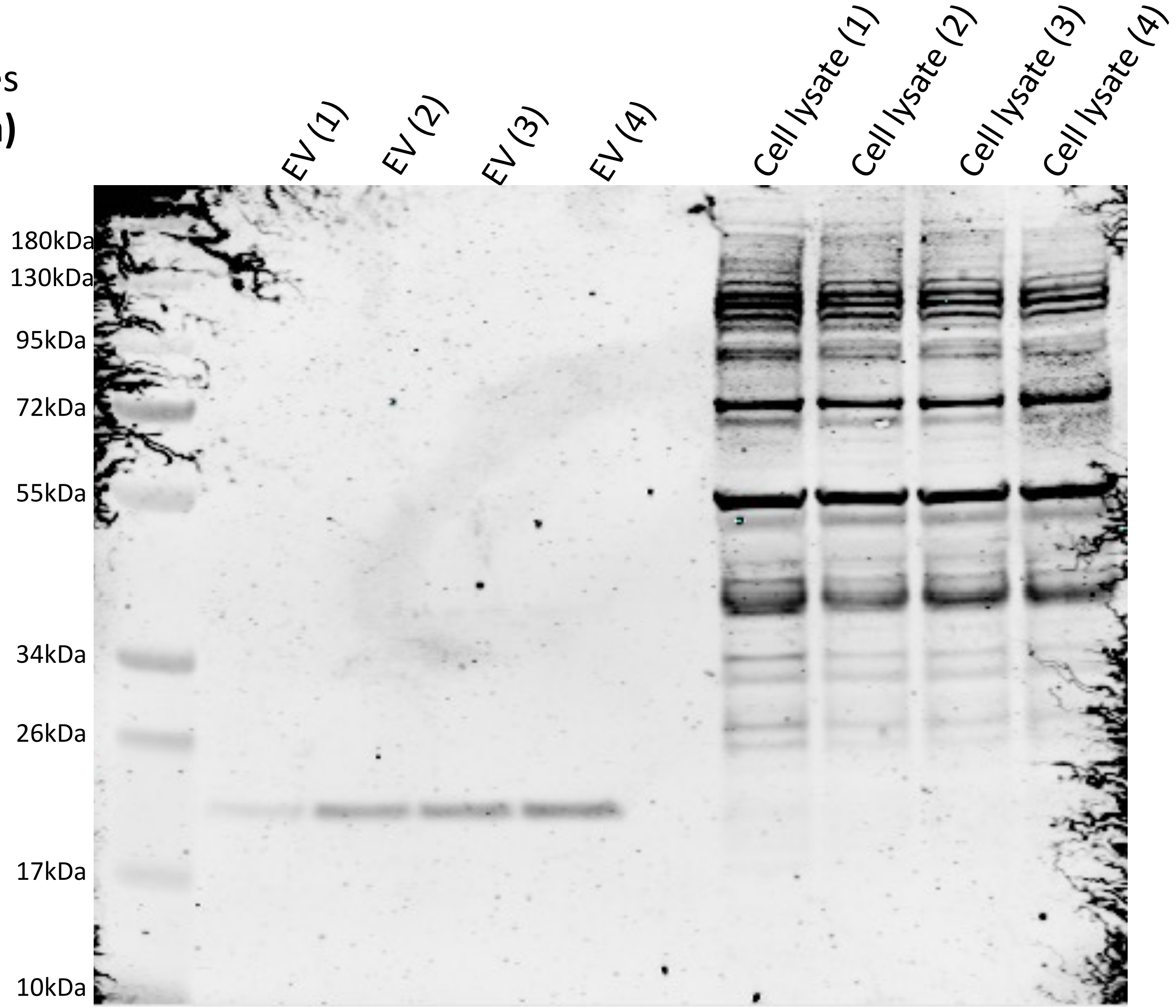

Raw Western Blot:
BV2 cells
EV lysates and cell lysates
Probed with TSG101 (49kDa)

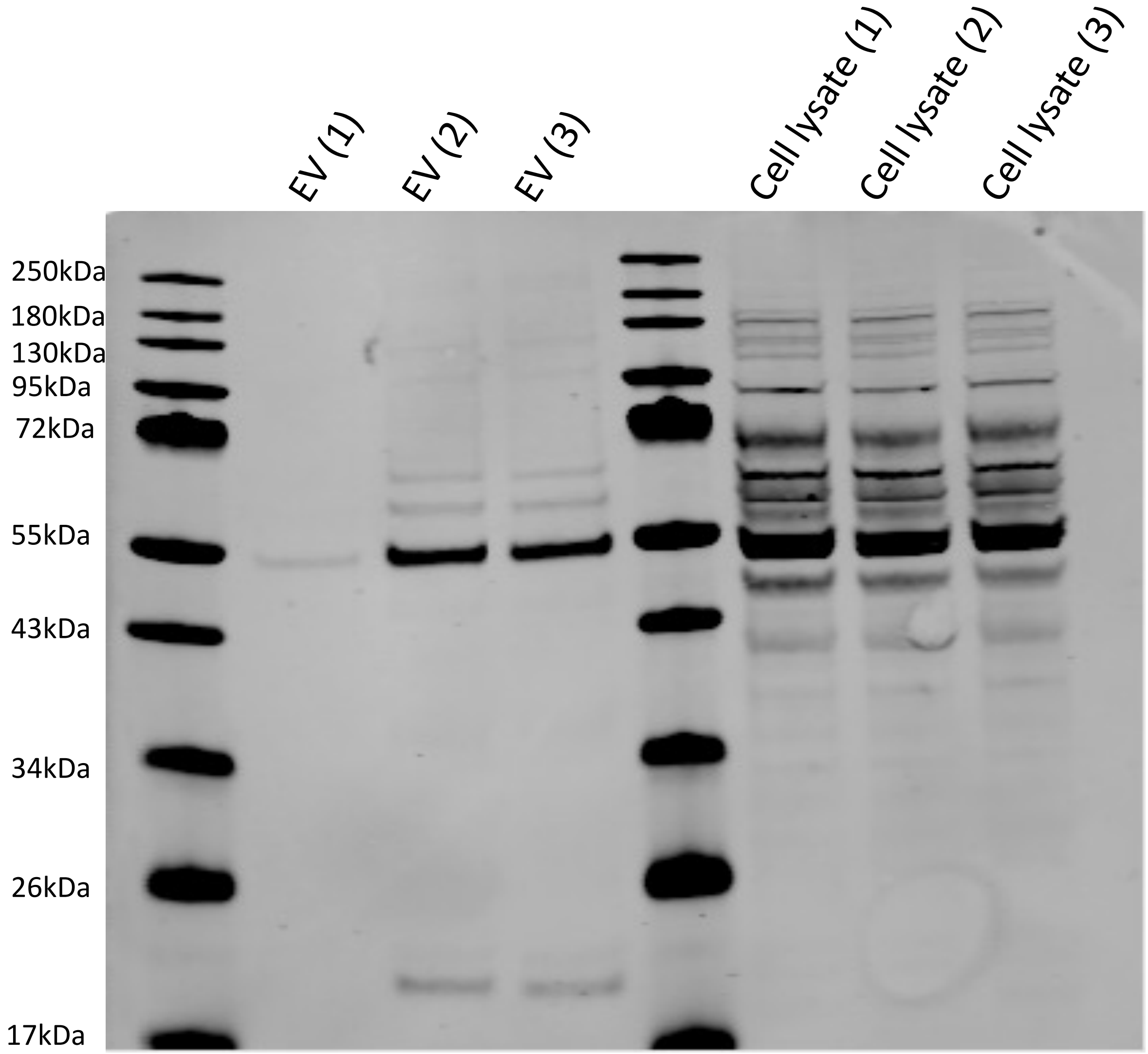

Raw western blot

BV2 cells

EV lysates and cell lysates

Probed with Calnexin (90Kda)

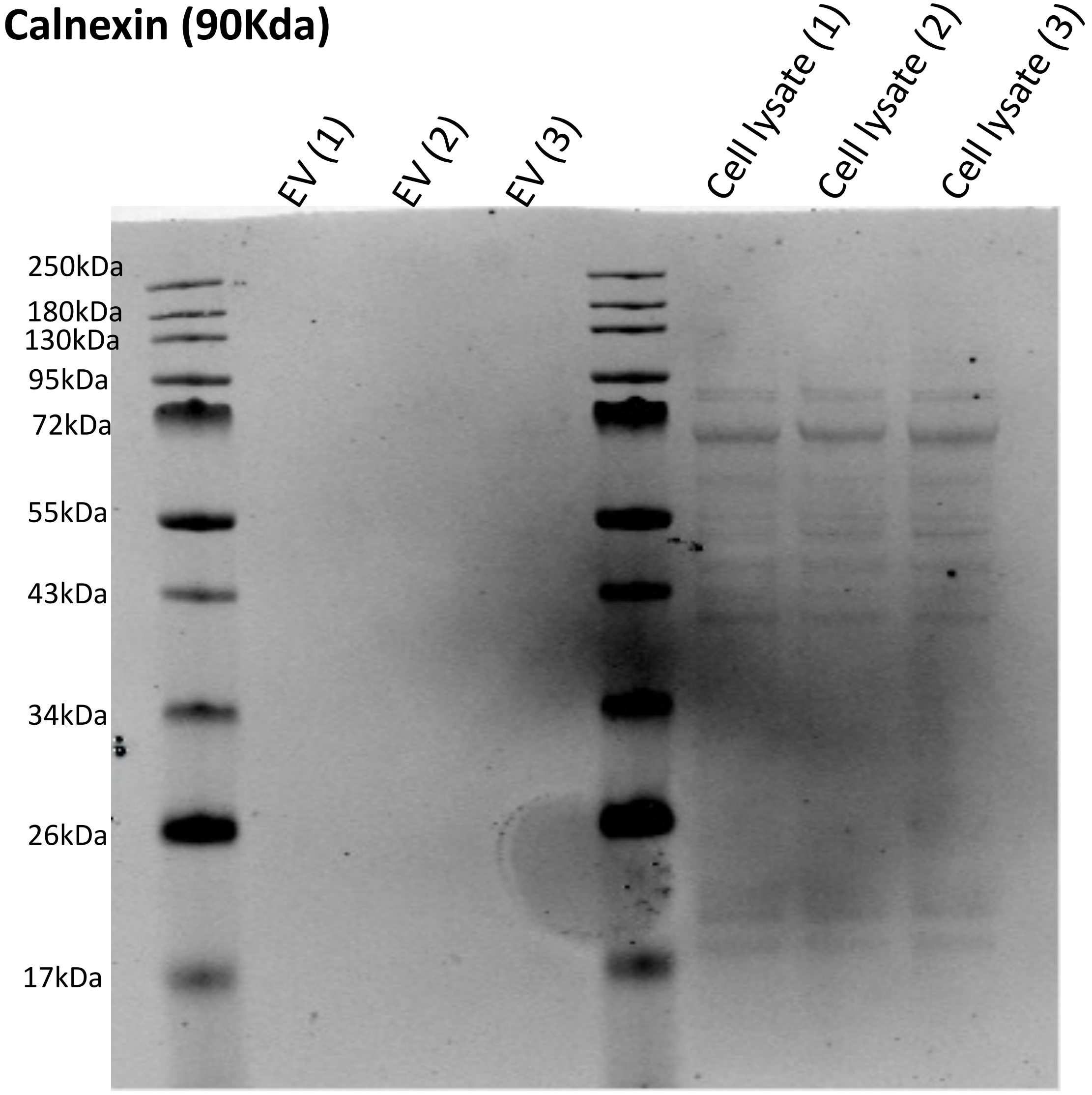

Supplement: Supplemental Data 1 [file mmc1.pdf]
