# Supplementary material for: Identification of State-Specific Proteomic and Transcriptomic Signatures of Microglia-Derived Extracellular Vesicles
Source: Mol Cell Proteomics. 2023 Nov 11;22(12):100678. doi: 10.1016/j.mcpro.2023.100678 (PMC10755493; doi:10.1016/j.mcpro.2023.100678)
Supplement: Supplemental Data 7 [file mmc7.pdf]

Sample ID Table & QC Results

Sample #	Admera Health Sample ID	Customer Sample ID	Sample Type	Sample Volume (ul)	Admera Health Concentration (ng/ul)	Admera Health Total Quantity (ng)	RIN
1	22082R-05-01	CTL Cell (1)	RNA	30	2800.0	84000	10.0
2	22082R-05-02	CTL Cell (2)	RNA	31	1690.0	52390	10.0
3	22082R-05-03	CTL Cell (3)	RNA	33	2380.0	78540	10.0
4	22082R-05-04	LPS Cell (1)	RNA	29	1130.0	32770	10.0
5	22082R-05-05	LPS Cell (2)	RNA	33	784.0	25872	10.0
6	22082R-05-06	LPS Cell (3)	RNA	33	1010.0	33330	10.0
7	22082R-05-07	IL-10 Cell (1)	RNA	32	1550.0	49600	10.0
8	22082R-05-08	IL-10 Cell (2)	RNA	32	1950.0	62400	10.0
9	22082R-05-09	IL-10 Cell (3)	RNA	33	1550.0	51150	10.0
10	22082R-05-10	TGFB Cell (1)	RNA	33	1680.0	55440	10.0
11	22082R-05-11	TGFB Cell (2)	RNA	33	2160.0	71280	10.0
12	22082R-05-12	TGFB Cell (3)	RNA	31	2160.0	66960	10.0
13	22082R-05-13	CTL EV (1)	RNA	18	0.1	2	N/A
14	22082R-05-14	CTL EV (2)	RNA	31	0.1	4	N/A
15	22082R-05-15	CTL EV (3)	RNA	18	0.2	4	N/A
16	22082R-05-16	LPS EV (1)	RNA	18	0.2	4	N/A
17	22082R-05-17	LPS EV (2)	RNA	18	0.9	16	2.0
18	22082R-05-18	LPS EV (3)	RNA	18	0.2	3	N/A
19	22082R-05-19	IL-10 EV (1)	RNA	18	0.1	2	N/A
20	22082R-05-20	IL-10 EV (2)	RNA	18	0.4	7	N/A
21	22082R-05-21	IL-10 EV (3)	RNA	18	0.3	6	N/A
22	22082R-05-22	TGFB EV (1)	RNA	18	0.3	5	N/A
23	22082R-05-23	TGFB EV (2)	RNA	18	0.3	5	N/A
24	22082R-05-24	TGFB EV (3)	RNA	18	0.2	4	N/A

TapeStation Image

Filename: 22082-05-01-12-IQC-TS-01252023.cRNA

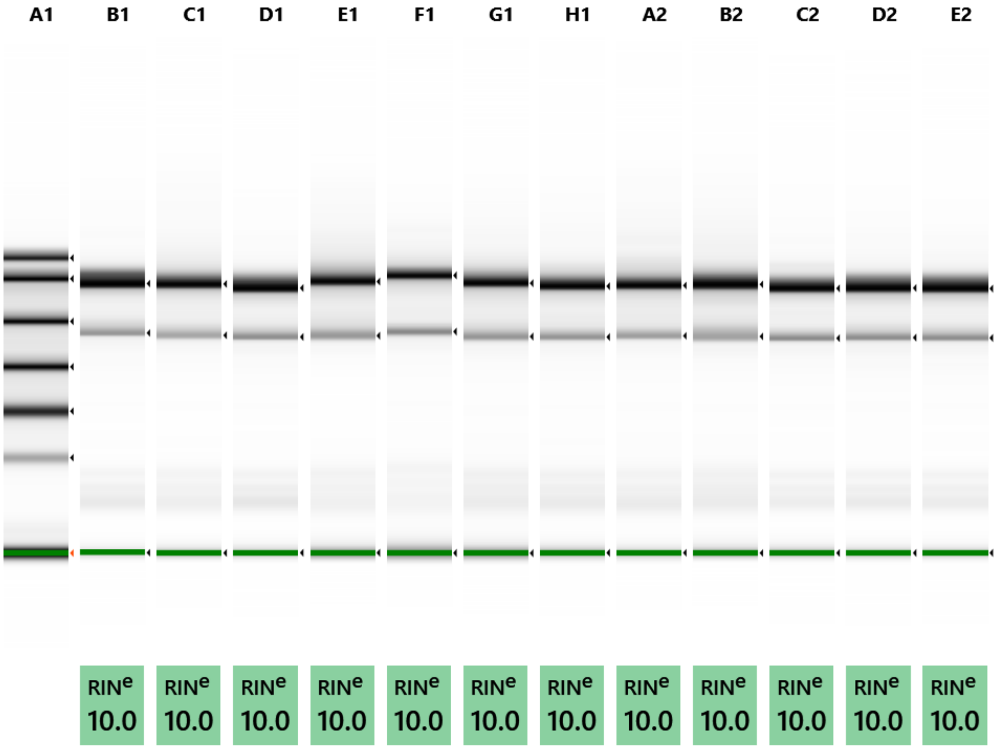

Default image (Contrast 50%), Image is Scaled to Sample

Sample Info

Well	RIN^e	28S/18S (Area)	Conc. [ng/µl]	Sample Description	Alert	Observations
A1	-	-	84.9	Electronic Ladder		Ladder
B1	10.0	2.4	318	22082R-05-01		
C1	10.0	3.2	199	22082R-05-02		
D1	10.0	2.0	229	22082R-05-03		
E1	10.0	2.7	118	22082R-05-04		
F1	10.0	2.5	74.2	22082R-05-05		
G1	10.0	2.6	108	22082R-05-06		
H1	10.0	3.0	157	22082R-05-07		
A2	10.0	3.1	233	22082R-05-08		
B2	10.0	2.7	176	22082R-05-09		
C2	10.0	3.1	179	22082R-05-10		
D2	10.0	3.2	228	22082R-05-11		
E2	10.0	3.3	238	22082R-05-12		

A1: Electronic Ladder

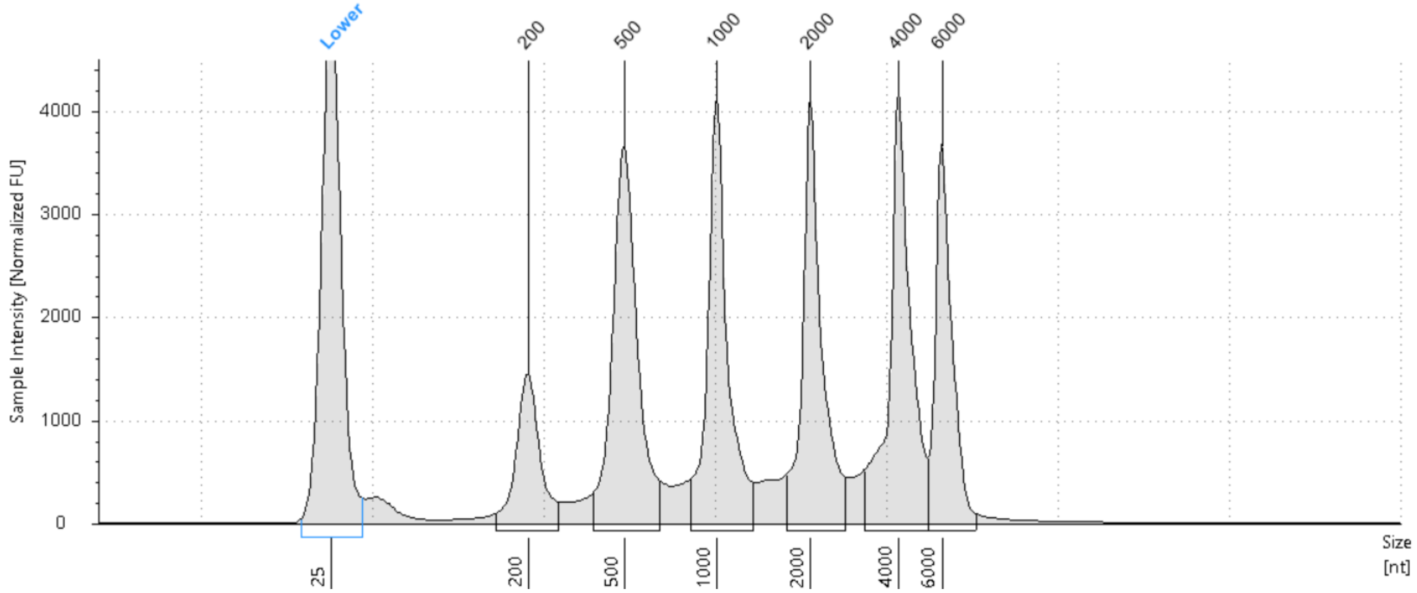

Sample Table

Well	RIN[e]	28S/18S (Area)	Conc. [ng/µl]	Sample Description	Alert	Observations
A1	-	-	84.9	Electronic Ladder		Ladder

Peak Table

Size [nt]	Calibrated Conc. [ng/µl]	Assigned Conc. [ng/µl]	Peak Molarity [nmol/l]	% Integrated Area	Peak Comment	Observations
25	40.0	40.0	4710	-		Lower Marker
200	5.94	-	87.4	7.80		
500	15.9	-	93.6	20.88		
1000	14.2	-	41.8	18.62		
2000	13.8	-	20.3	18.11		
4000	15.5	-	11.4	20.38		
6000	10.8	-	5.31	14.22		

B1: 22082R-05-01

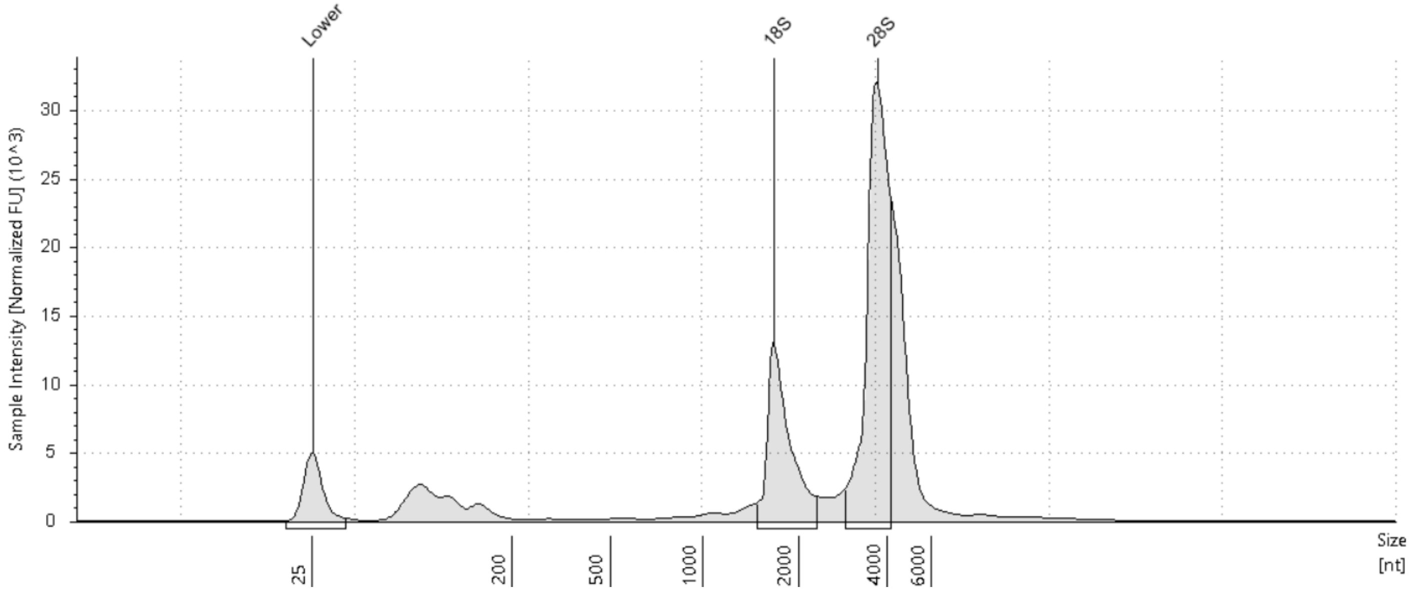

Sample Table

Well	RIN[e]	28S/18S (Area)	Conc. [ng/µl]	Sample Description	Alert	Observations
B1	10.0	2.4	318	22082R-05-01		

Peak Table

Size [nt]	Calibrated Conc. [ng/µl]	Assigned Conc. [ng/µl]	Peak Molarity [nmol/l]	% Integrated Area	Peak Comment	Observations
25	40.0	40.0	4710	-		Lower Marker
1668	57.2	-	101	29.80		18S
3683	135	-	108	70.20		28S

C1: 22082R-05-02

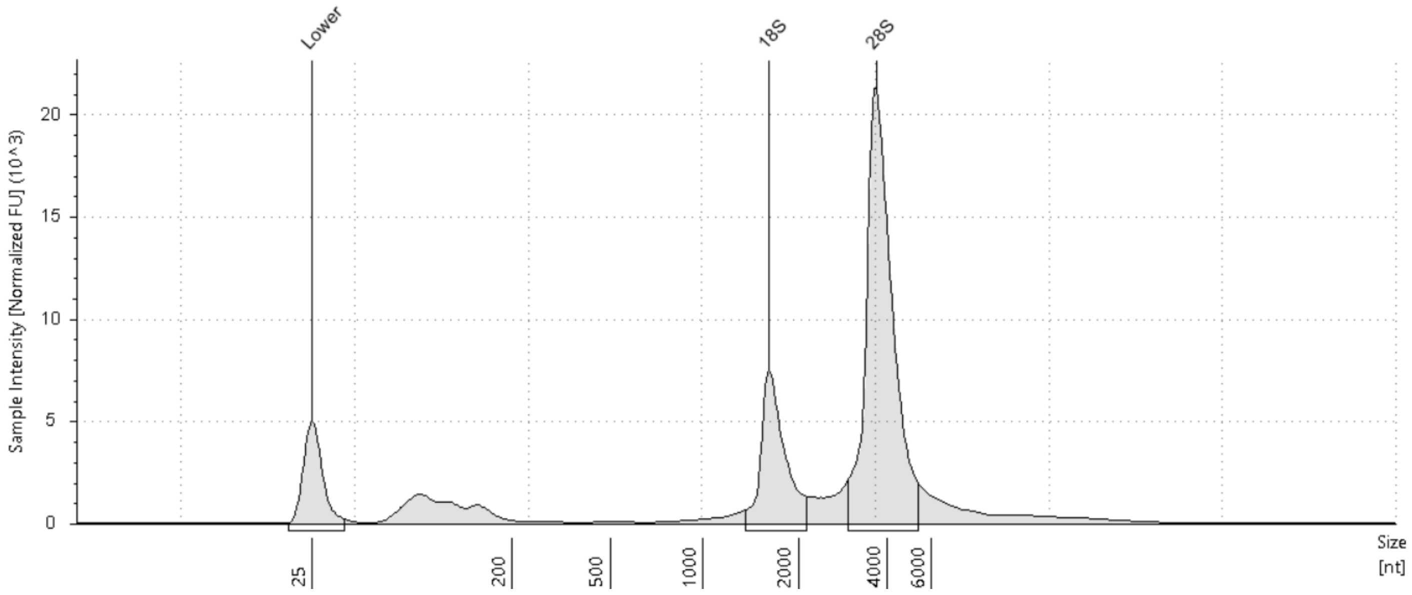

Sample Table

Well	RIN^e	28S/18S (Area)	Conc. [ng/µl]	Sample Description	Alert	Observations
C1	10.0	3.2	199	22082R-05-02		

Peak Table

Size [nt]	Calibrated Conc. [ng/µl]	Assigned Conc. [ng/µl]	Peak Molarity [nmol/l]	% Integrated Area	Peak Comment	Observations
25	40.0	40.0	4710	-		Lower Marker
1616	34.9	-	63.5	23.98		18S
3671	111	-	88.6	76.02		28S

D1: 22082R-05-03

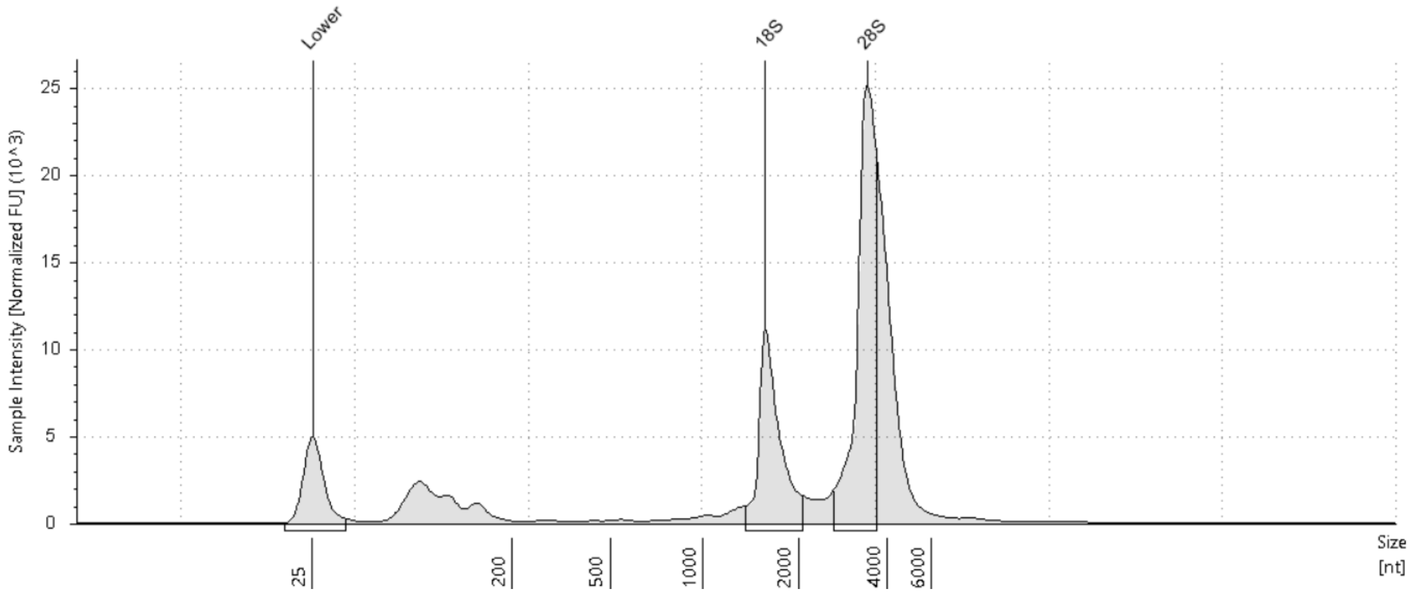

Sample Table

Well	RIN^e	28S/18S (Area)	Conc. [ng/µl]	Sample Description	Alert	Observations
D1	10.0	2.0	229	22082R-05-03		

Peak Table

Size [nt]	Calibrated Conc. [ng/µl]	Assigned Conc. [ng/µl]	Peak Molarity [nmol/l]	% Integrated Area	Peak Comment	Observations
25	40.0	40.0	4710	-		Lower Marker
1570	42.3	-	79.3	33.29		18S
3424	84.8	-	72.9	66.71		28S

E1: 22082R-05-04

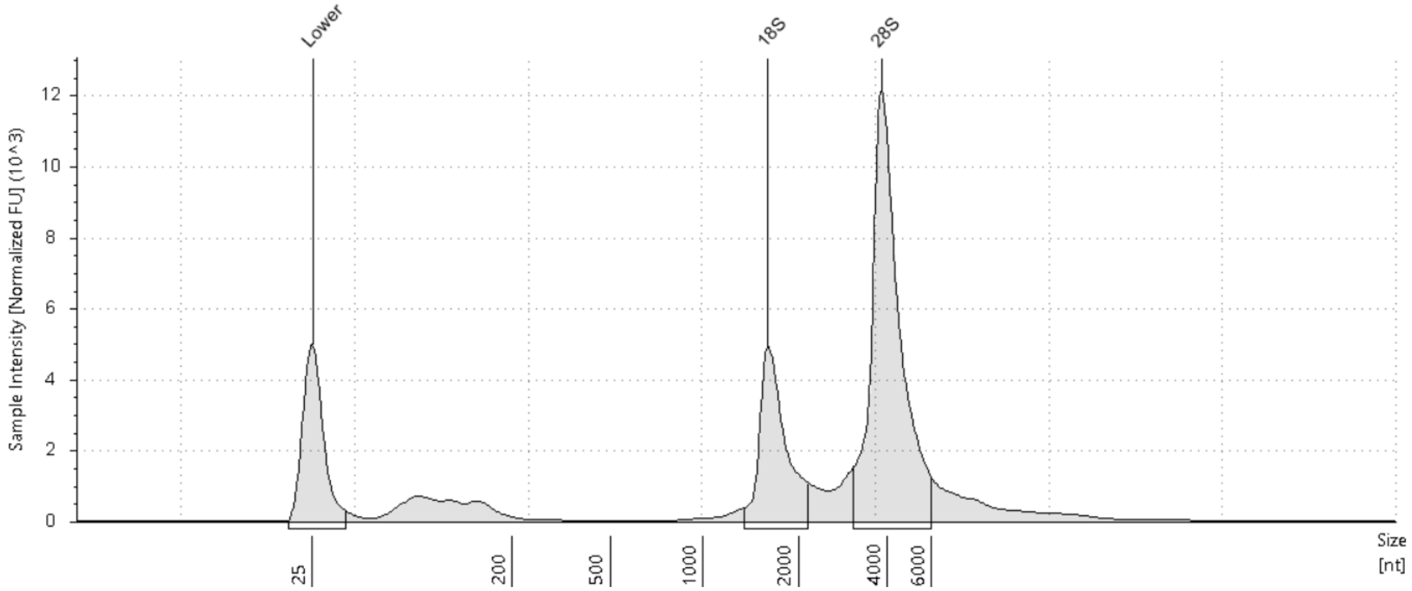

Sample Table

Well	RIN^e	28S/18S (Area)	Conc. [ng/µl]	Sample Description	Alert	Observations
E1	10.0	2.7	118	22082R-05-04		

Peak Table

Size [nt]	Calibrated Conc. [ng/µl]	Assigned Conc. [ng/µl]	Peak Molarity [nmol/l]	% Integrated Area	Peak Comment	Observations
25	40.0	40.0	4710	-		Lower Marker
1606	23.3	-	42.7	27.33		18S
3822	62.0	-	47.7	72.67		28S

F1: 22082R-05-05

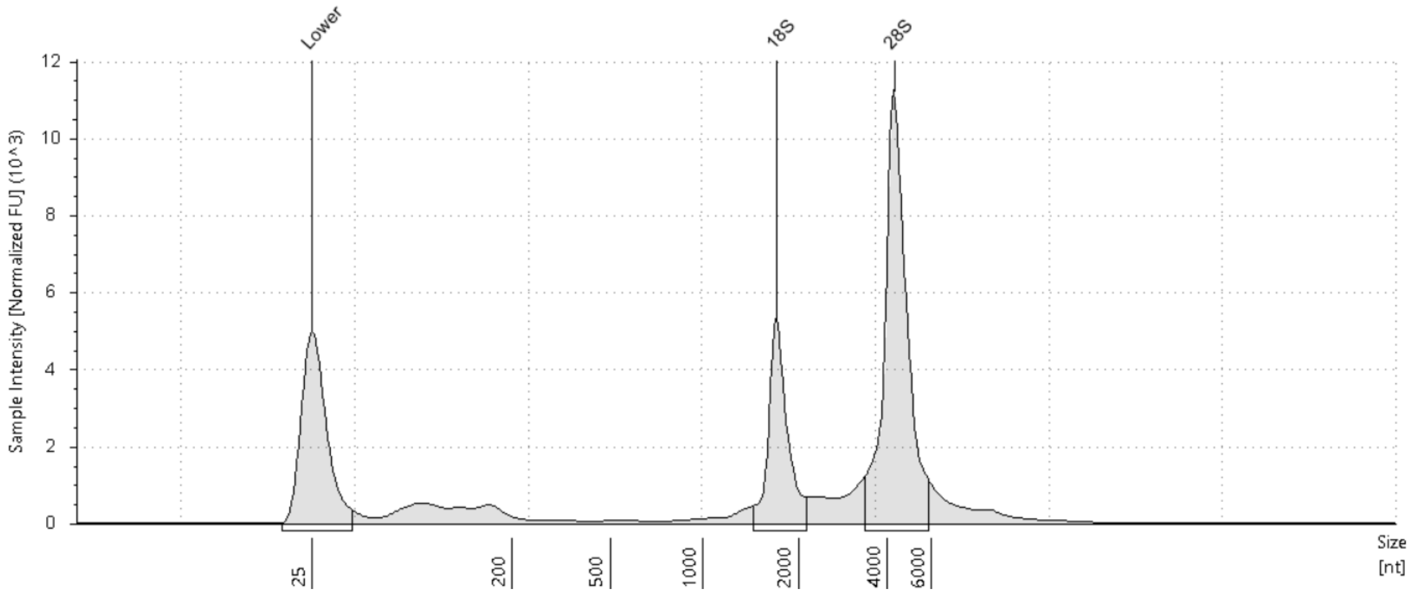

Sample Table

Well	RIN^e	28S/18S (Area)	Conc. [ng/µl]	Sample Description	Alert	Observations
F1	10.0	2.5	74.2	22082R-05-05		

Peak Table

Size [nt]	Calibrated Conc. [ng/µl]	Assigned Conc. [ng/µl]	Peak Molarity [nmol/l]	% Integrated Area	Peak Comment	Observations
25	40.0	40.0	4710	-		Lower Marker
1711	14.7	-	25.4	28.55		18S
4265	36.9	-	25.5	71.45		28S

G1: 22082R-05-06

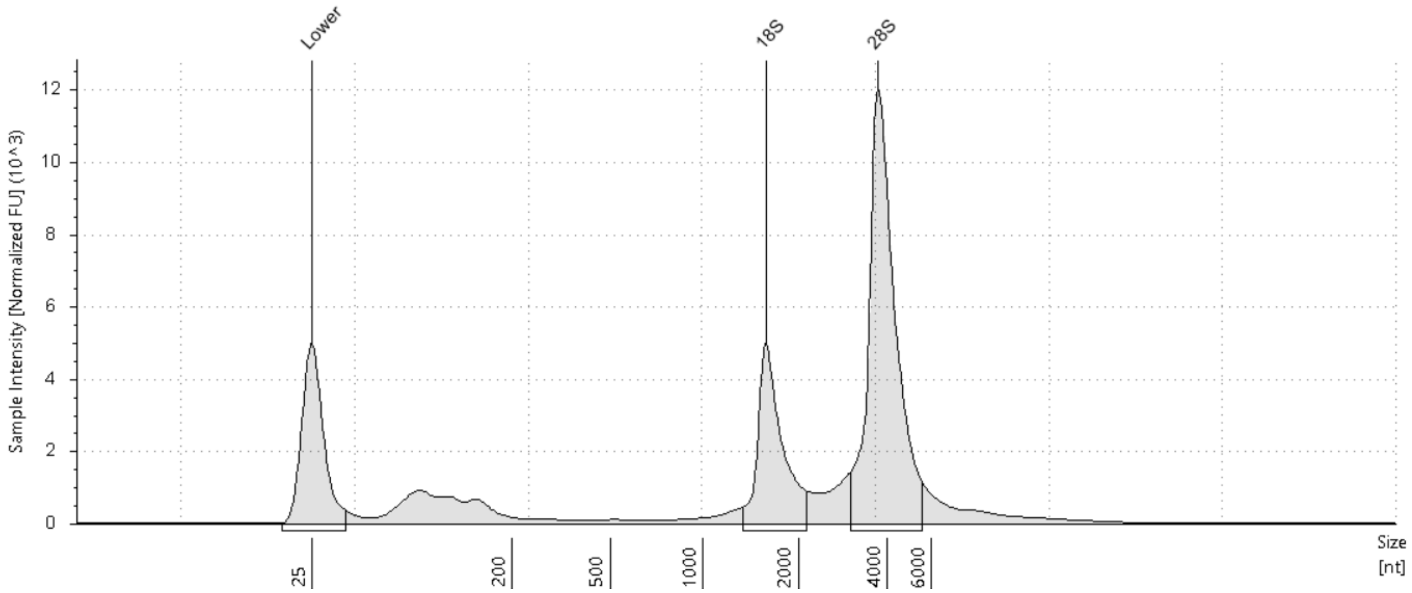

Sample Table

Well	RIN^e	28S/18S (Area)	Conc. [ng/µl]	Sample Description	Alert	Observations
G1	10.0	2.6	108	22082R-05-06		

Peak Table

Size [nt]	Calibrated Conc. [ng/µl]	Assigned Conc. [ng/µl]	Peak Molarity [nmol/l]	% Integrated Area	Peak Comment	Observations
25	40.0	40.0	4710	-		Lower Marker
1590	21.0	-	38.9	27.53		18S
3730	55.4	-	43.6	72.47		28S

H1: 22082R-05-07

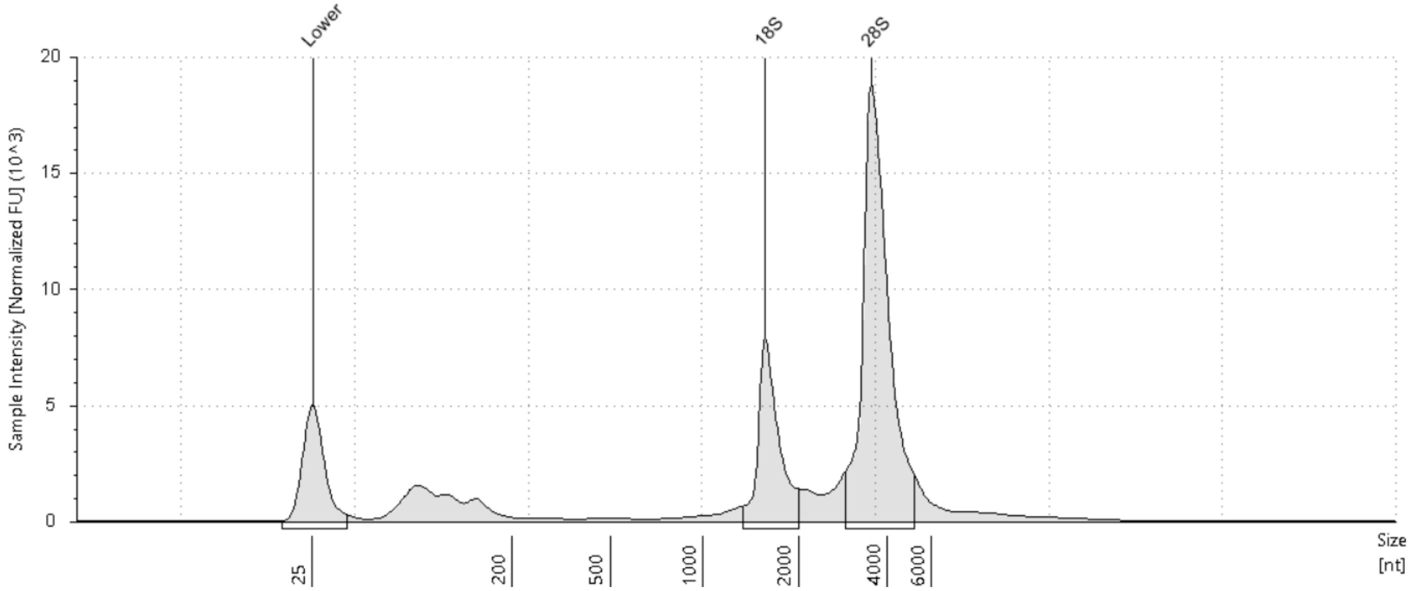

Sample Table

Well	RIN^e	28S/18S (Area)	Conc. [ng/µl]	Sample Description	Alert	Observations
H1	10.0	3.0	157	22082R-05-07		

Peak Table

Size [nt]	Calibrated Conc. [ng/µl]	Assigned Conc. [ng/µl]	Peak Molarity [nmol/l]	% Integrated Area	Peak Comment	Observations
25	40.0	40.0	4710	-		Lower Marker
1571	27.9	-	52.2	24.94		18S
3536	84.0	-	69.9	75.06		28S

A2: 22082R-05-08

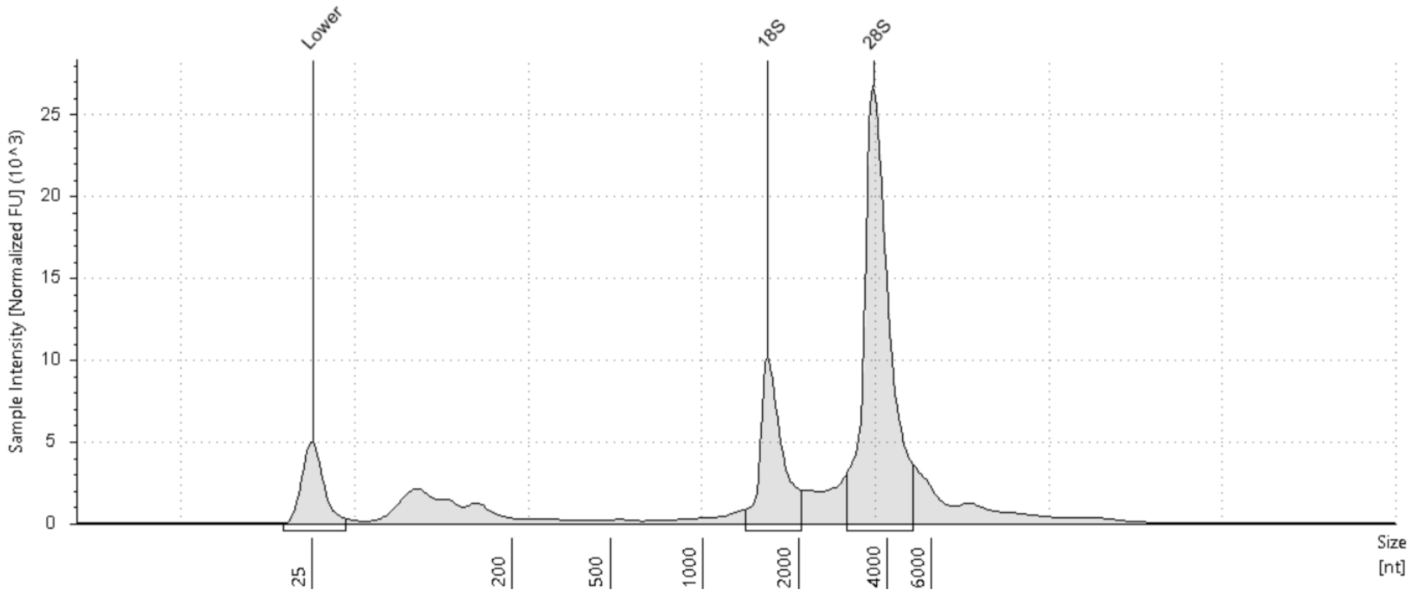

Sample Table

Well	RIN^{e}	28S/18S (Area)	Conc. [ng/µl]	Sample Description	Alert	Observations
A2	10.0	3.1	233	22082R-05-08		

Peak Table

Size [nt]	Calibrated Conc. [ng/µl]	Assigned Conc. [ng/µl]	Peak Molarity [nmol/l]	% Integrated Area	Peak Comment	Observations
25	40.0	40.0	4710	-		Lower Marker
1600	38.1	-	70.0	24.47		18S
3575	118	-	96.7	75.53		28S

B2: 22082R-05-09

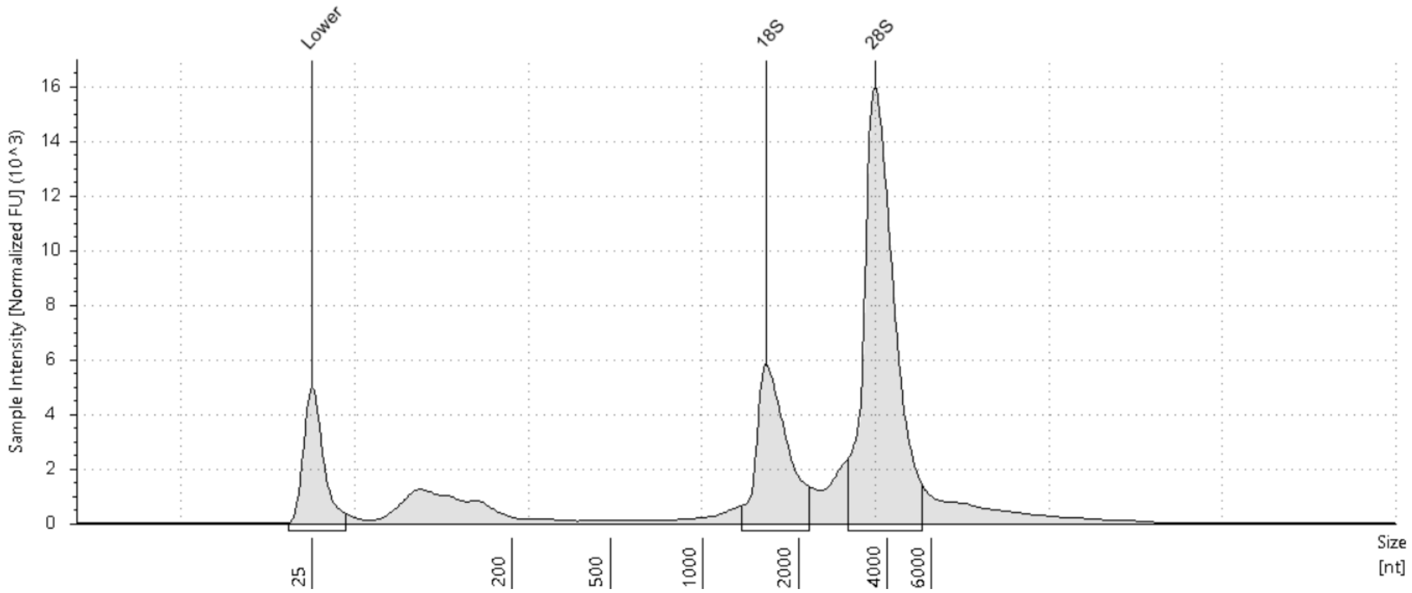

Sample Table

Well	RIN[e]	28S/18S (Area)	Conc. [ng/µl]	Sample Description	Alert	Observations
B2	10.0	2.7	176	22082R-05-09		

Peak Table

Size [nt]	Calibrated Conc. [ng/µl]	Assigned Conc. [ng/µl]	Peak Molarity [nmol/l]	% Integrated Area	Peak Comment	Observations
25	40.0	40.0	4710	-		Lower Marker
1590	33.6	-	62.1	26.72		18S
3659	92.2	-	74.1	73.28		28S

C2: 22082R-05-10

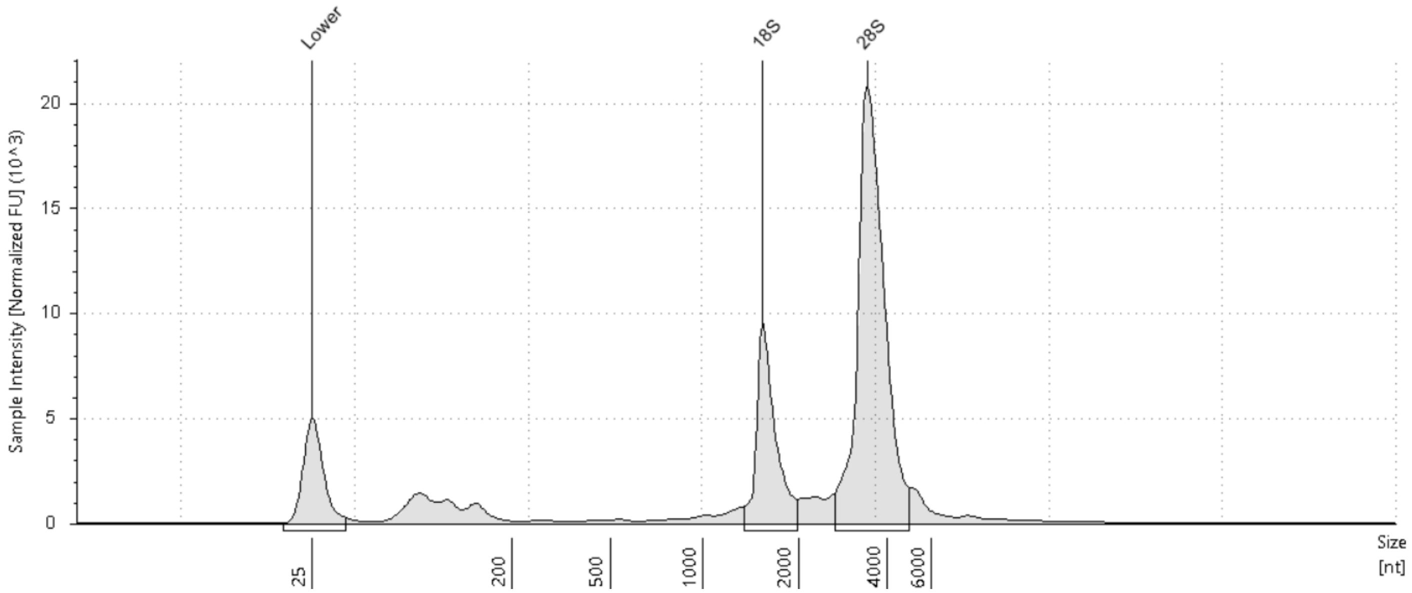

Sample Table

Well	RIN^e	28S/18S (Area)	Conc. [ng/µl]	Sample Description	Alert	Observations
C2	10.0	3.1	179	22082R-05-10		

Peak Table

Size [nt]	Calibrated Conc. [ng/µl]	Assigned Conc. [ng/µl]	Peak Molarity [nmol/l]	% Integrated Area	Peak Comment	Observations
25	40.0	40.0	4710	-		Lower Marker
1545	33.0	-	62.8	24.14		18S
3417	104	-	89.2	75.86		28S

D2: 22082R-05-11

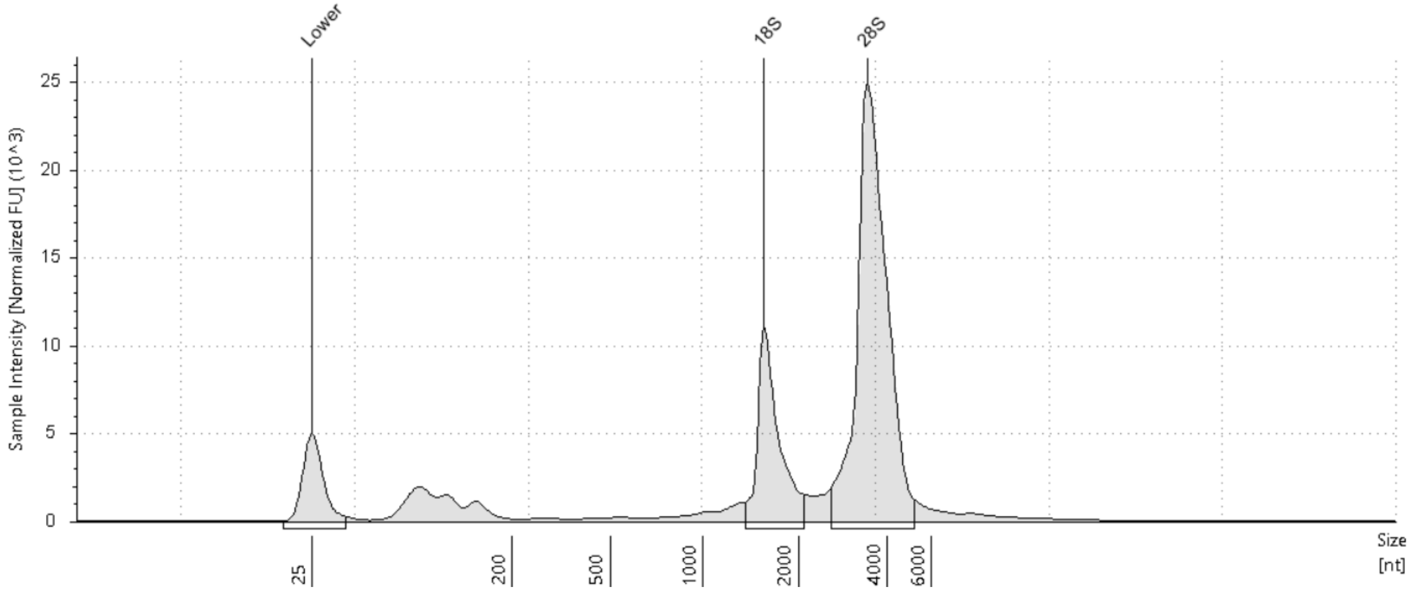

Sample Table

Well	RIN^e	28S/18S (Area)	Conc. [ng/µl]	Sample Description	Alert	Observations
D2	10.0	3.2	228	22082R-05-11		

Peak Table

Size [nt]	Calibrated Conc. [ng/µl]	Assigned Conc. [ng/µl]	Peak Molarity [nmol/l]	% Integrated Area	Peak Comment	Observations
25	40.0	40.0	4710	-		Lower Marker
1562	42.6	-	80.2	23.80		18S
3442	136	-	116	76.20		28S

E2: 22082R-05-12

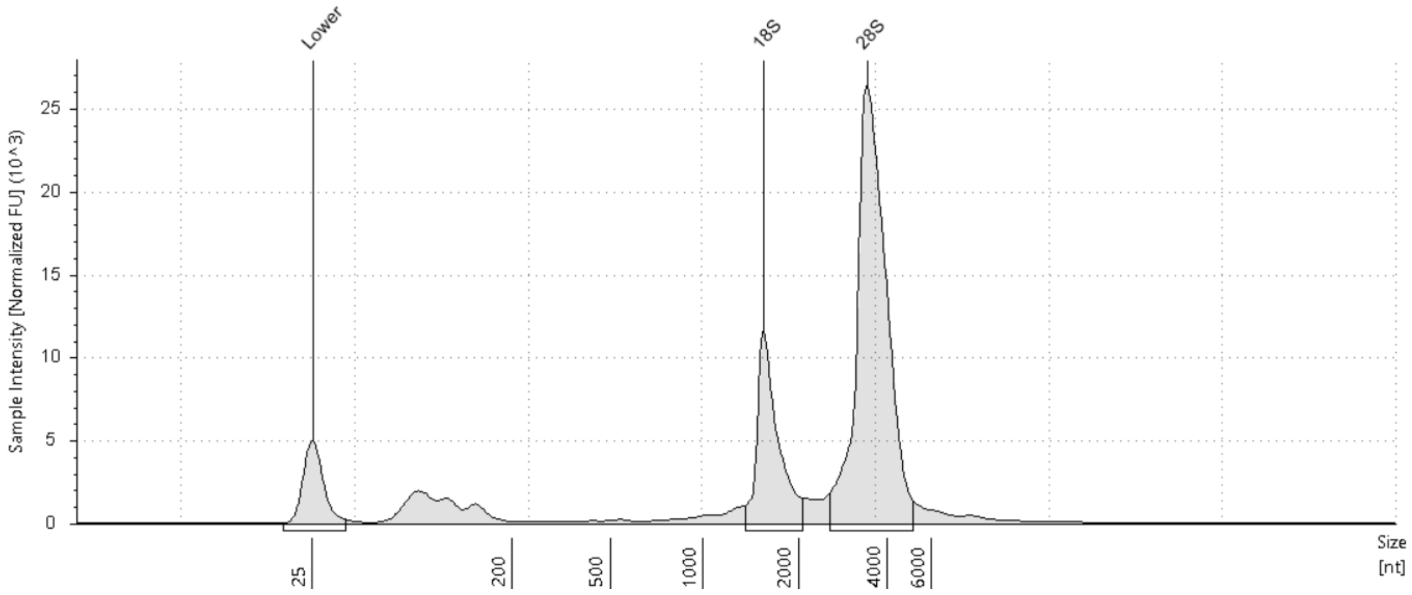

Sample Table

Well	RIN^e	28S/18S (Area)	Conc. [ng/µl]	Sample Description	Alert	Observations
E2	10.0	3.3	238	22082R-05-12		

Peak Table

Size [nt]	Calibrated Conc. [ng/µl]	Assigned Conc. [ng/µl]	Peak Molarity [nmol/l]	% Integrated Area	Peak Comment	Observations
25	40.0	40.0	4710	-		Lower Marker
1550	44.5	-	84.5	23.44		18S
3401	146	-	126	76.56		28S

Filename: 22082-05-13-24-IQC-TS-01252023.cHSRNA

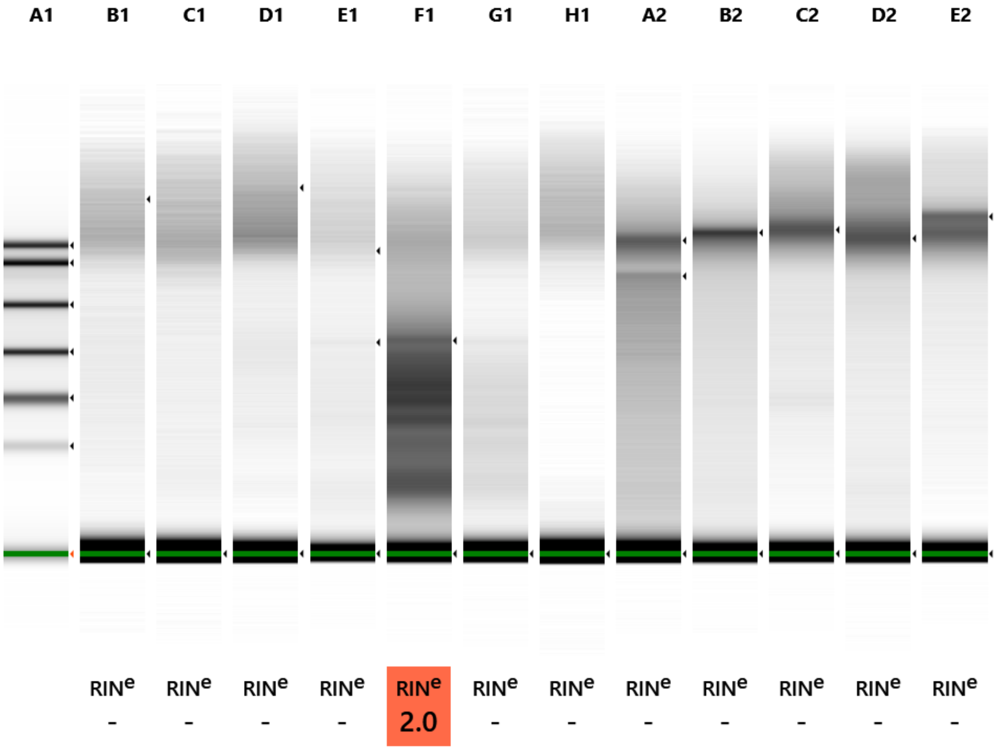

Default image (Contrast 50%), Image is Scaled to Sample

Sample Info

Well	RINe	28S/18S (Area)	Conc. [pg/µl]	Sample Description	Alert	Observations
A1	-	-	3750	Electronic Ladder		Ladder
B1	-	-	135	22082R-05-13		
C1	-	-	129	22082R-05-14		
D1	-	-	198	22082R-05-15		
E1	-	-	212	22082R-05-16		
F1	2.0	-	906	22082R-05-17		
G1	-	-	179	22082R-05-18		
H1	-	-	87.4	22082R-05-19		
A2	-	-	373	22082R-05-20		
B2	-	-	321	22082R-05-21		
C2	-	-	282	22082R-05-22		
D2	-	-	281	22082R-05-23		
E2	-	-	238	22082R-05-24		

A1: Electronic Ladder

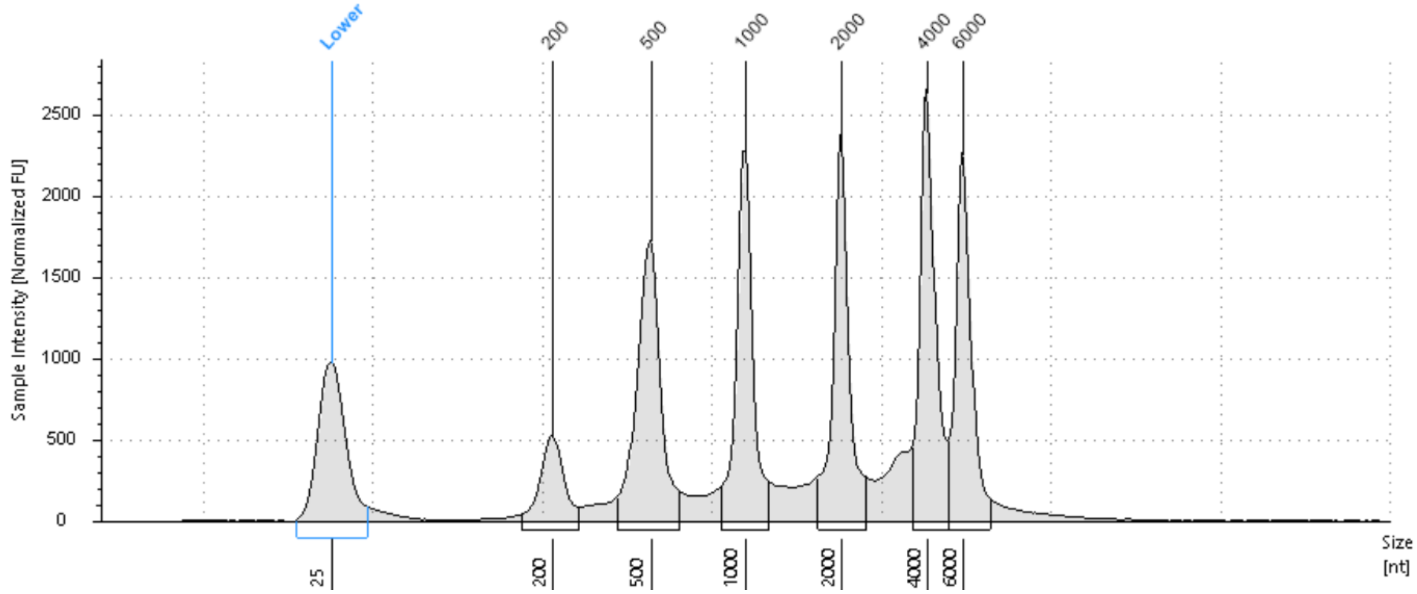

Sample Table

Well	RIN^e	28S/18S (Area)	Conc. [pg/µl]	Sample Description	Alert	Observations
A1	-	-	3750	Electronic Ladder		Ladder

Peak Table

Size [nt]	Calibrated Conc. [pg/µl]	Assigned Conc. [pg/µl]	Peak Molarity [pmol/l]	% Integrated Area	Peak Comment	Observations
25	700	700	82400	-		Lower Marker
200	189	-	2770	5.98		
500	638	-	3760	20.25		
1000	557	-	1640	17.66		
2000	580	-	854	18.42		
4000	634	-	466	20.11		
6000	554	-	272	17.58		

B1: 22082R-05-13

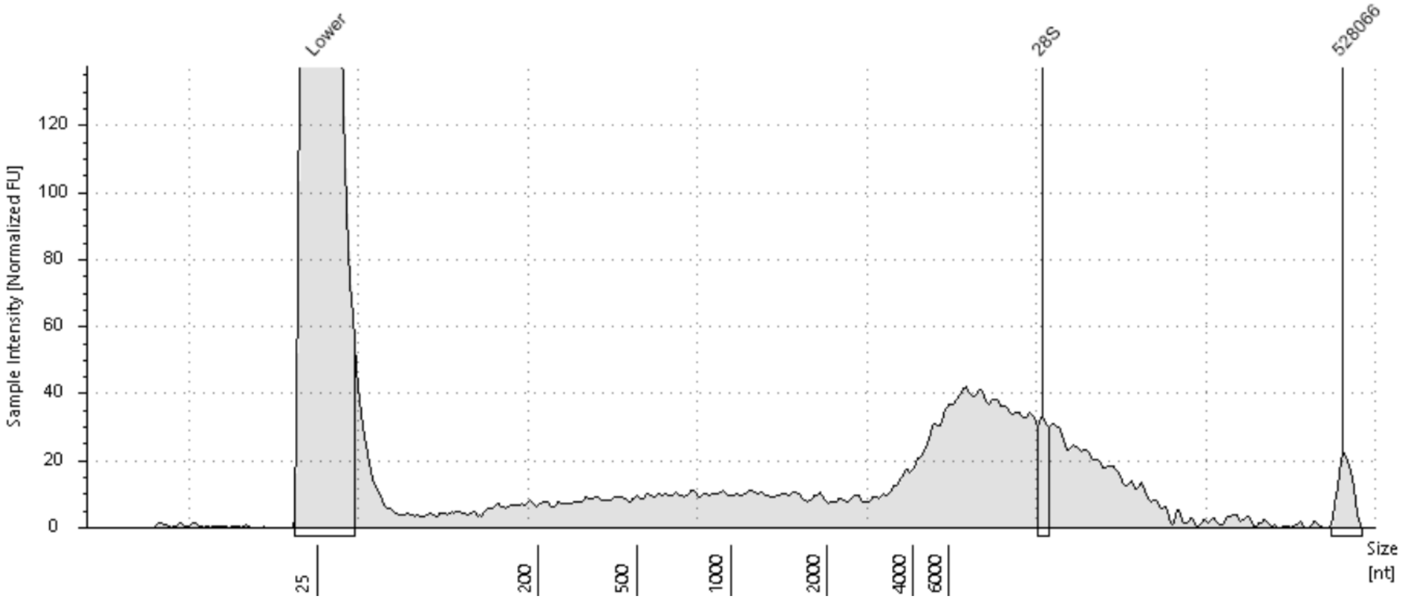

Sample Table

Well	RIN[e]	28S/18S (Area)	Conc. [pg/µl]	Sample Description	Alert	Observations
B1	-	-	135	22082R-05-13		

Peak Table

Size [nt]	Calibrated Conc. [pg/µl]	Assigned Conc. [pg/µl]	Peak Molarity [pmol/l]	% Integrated Area	Peak Comment	Observations
25	700	700	82400	-		Lower Marker
17500	4.07	-	0.685	49.71		28S
528066	4.12	-	0.0230	50.29		

C1: 22082R-05-14

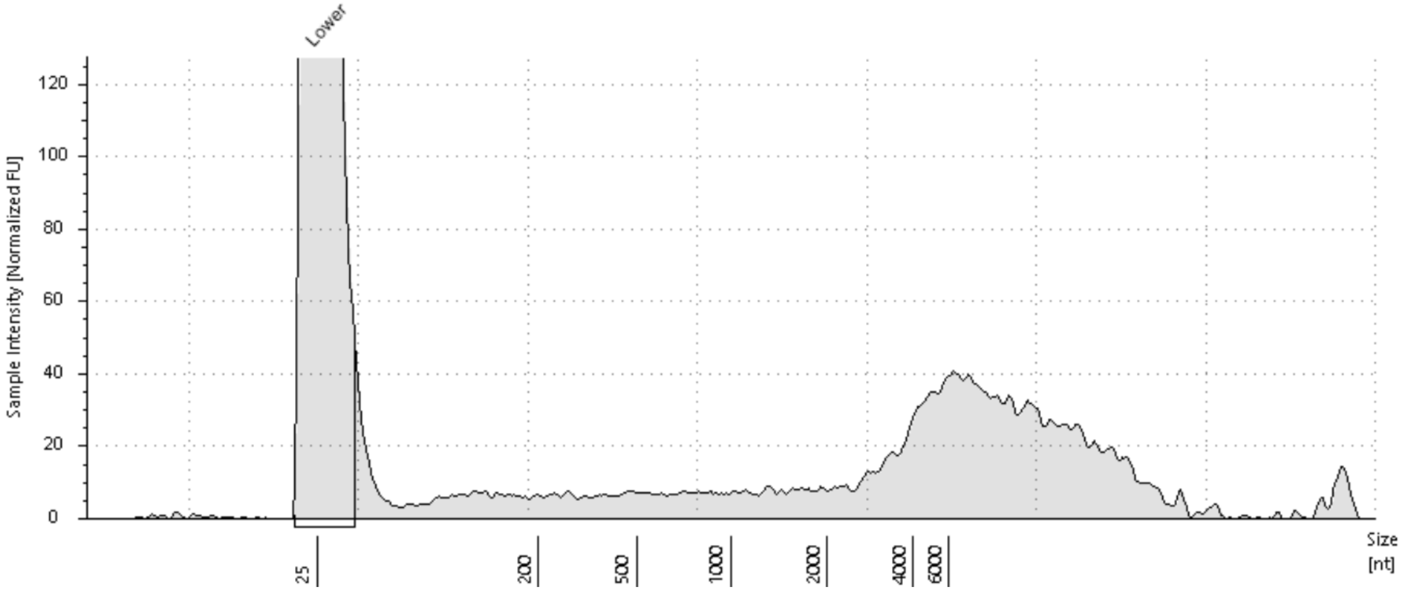

Sample Table

Well	RIN[e]	28S/18S (Area)	Conc. [pg/µl]	Sample Description	Alert	Observations
C1	-	-	129	22082R-05-14		

Peak Table

Size [nt]	Calibrated Conc. [pg/µl]	Assigned Conc. [pg/µl]	Peak Molarity [pmol/l]	% Integrated Area	Peak Comment	Observations
25	700	700	82400	-		Lower Marker

D1: 22082R-05-15

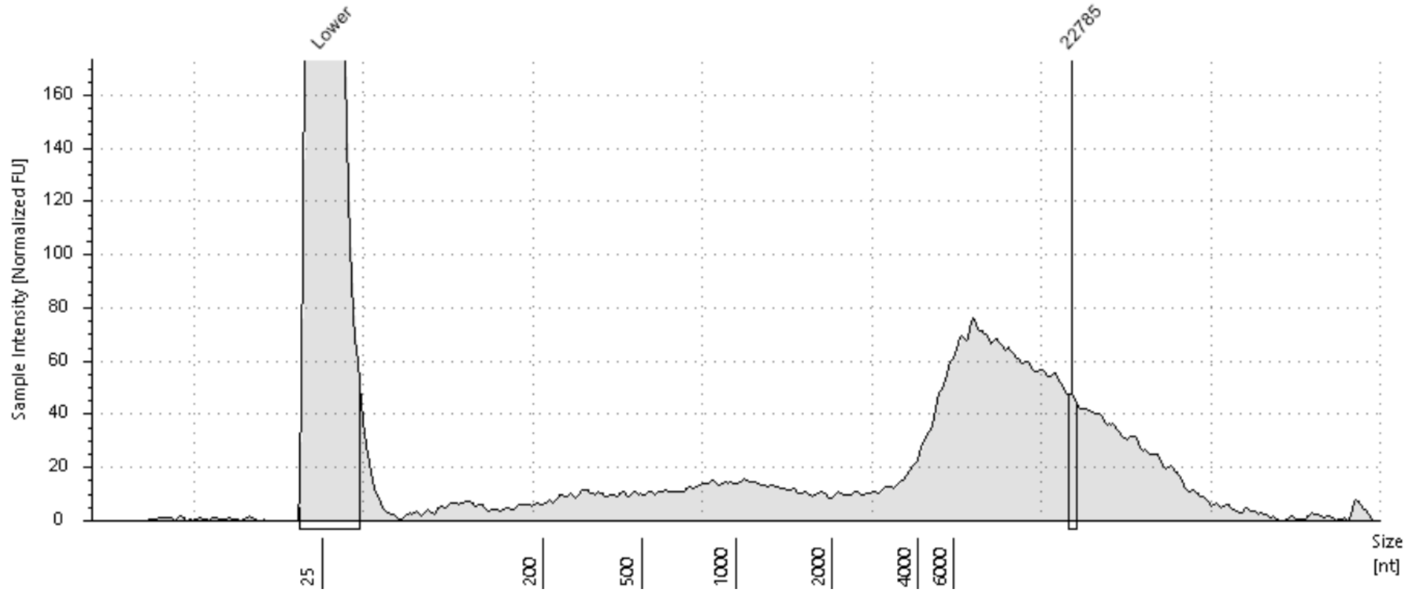

Sample Table

Well	RINe	28S/18S (Area)	Conc. [pg/µl]	Sample Description	Alert	Observations
D1	-	-	198	22082R-05-15		

Peak Table

Size [nt]	Calibrated Conc. [pg/µl]	Assigned Conc. [pg/µl]	Peak Molarity [pmol/l]	% Integrated Area	Peak Comment	Observations
25	700	700	82400	-		Lower Marker
22785	4.74	-	0.612	100.00		

E1: 22082R-05-16

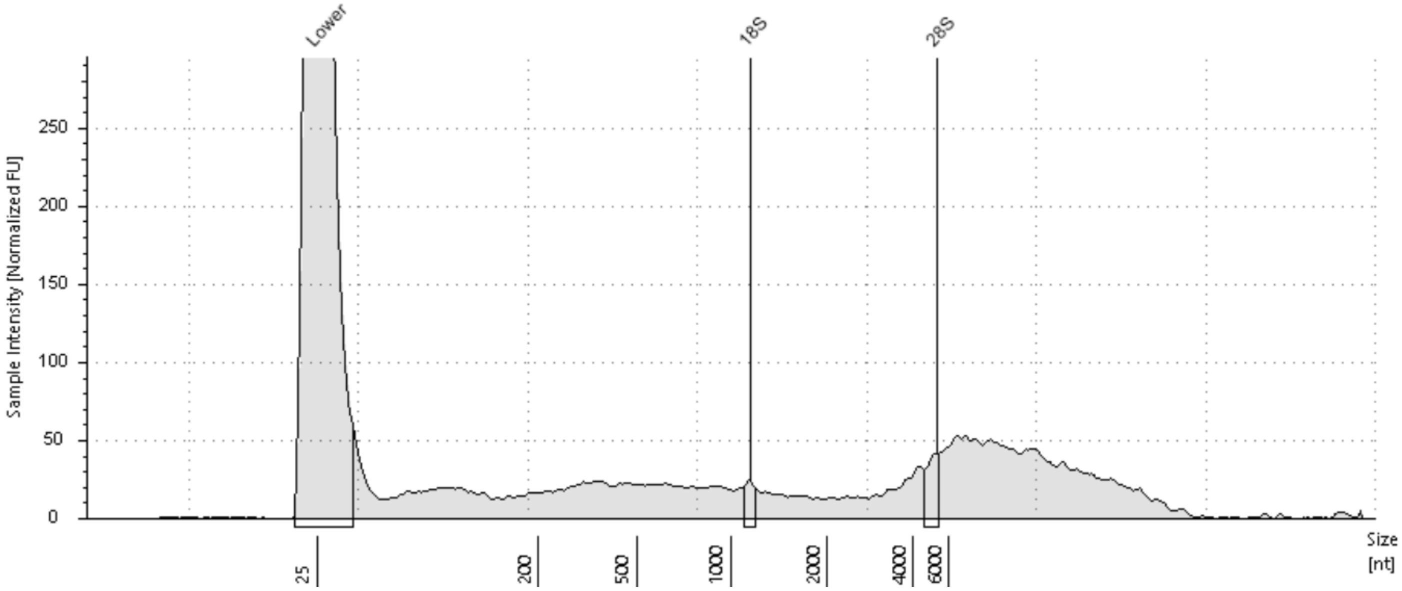

Sample Table

Well	RIN^e	28S/18S (Area)	Conc. [pg/µl]	Sample Description	Alert	Observations
E1	-	-	212	22082R-05-16		

Peak Table

Size [nt]	Calibrated Conc. [pg/µl]	Assigned Conc. [pg/µl]	Peak Molarity [pmol/l]	% Integrated Area	Peak Comment	Observations
25	700	700	82400	-		Lower Marker
1142	3.09	-	7.95	31.19		18S
5228	6.81	-	3.83	68.81		28S

F1: 22082R-05-17

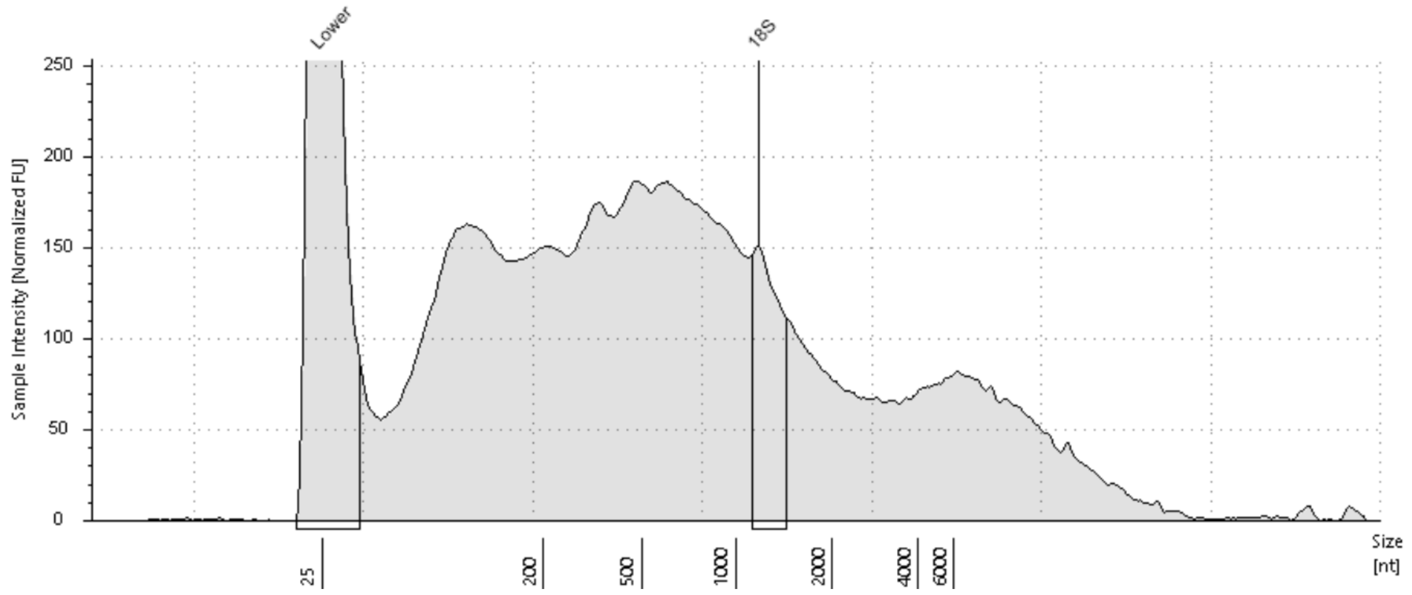

Sample Table

Well	RIN^e	28S/18S (Area)	Conc. [pg/µl]	Sample Description	Alert	Observations
F1	2.0	-	906	22082R-05-17		

Peak Table

Size [nt]	Calibrated Conc. [pg/µl]	Assigned Conc. [pg/µl]	Peak Molarity [pmol/l]	% Integrated Area	Peak Comment	Observations
25	700	700	82400	-		Lower Marker
1176	50.3	-	126	100.00		18S

G1: 22082R-05-18

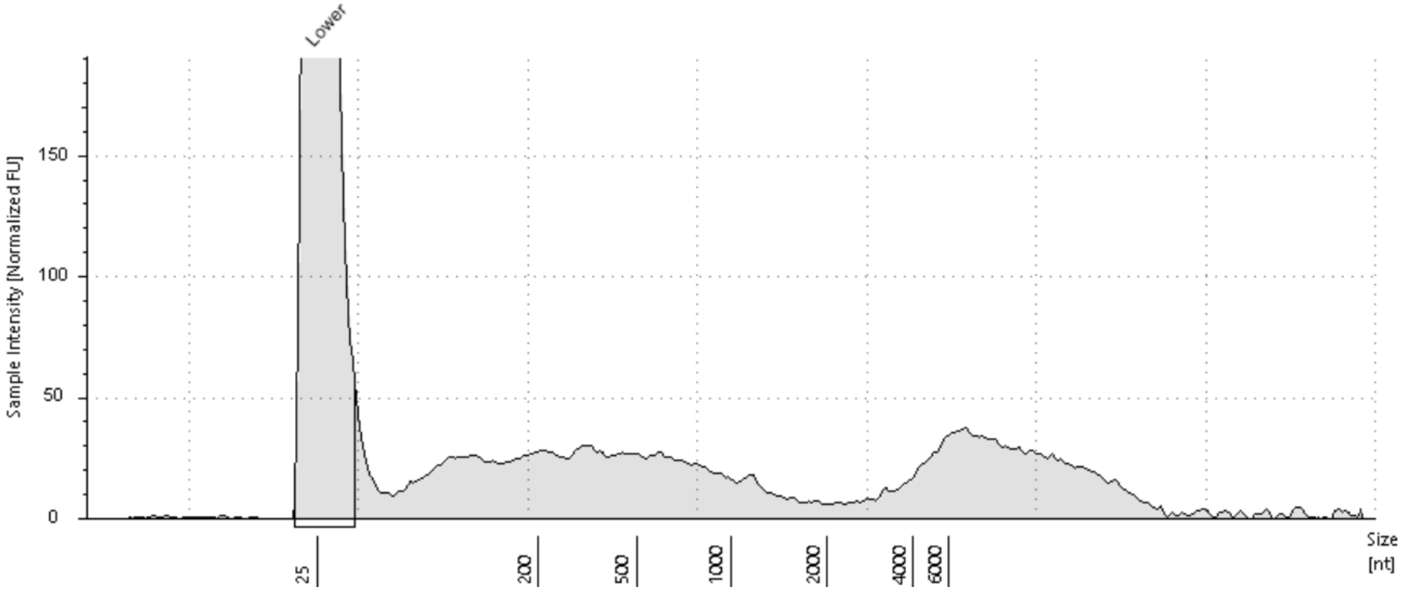

Sample Table

Well	RIN^e	28S/18S (Area)	Conc. [pg/µl]	Sample Description	Alert	Observations
G1	-	-	179	22082R-05-18		

Peak Table

Size [nt]	Calibrated Conc. [pg/µl]	Assigned Conc. [pg/µl]	Peak Molarity [pmol/l]	% Integrated Area	Peak Comment	Observations
25	700	700	82400	-		Lower Marker

H1: 22082R-05-19

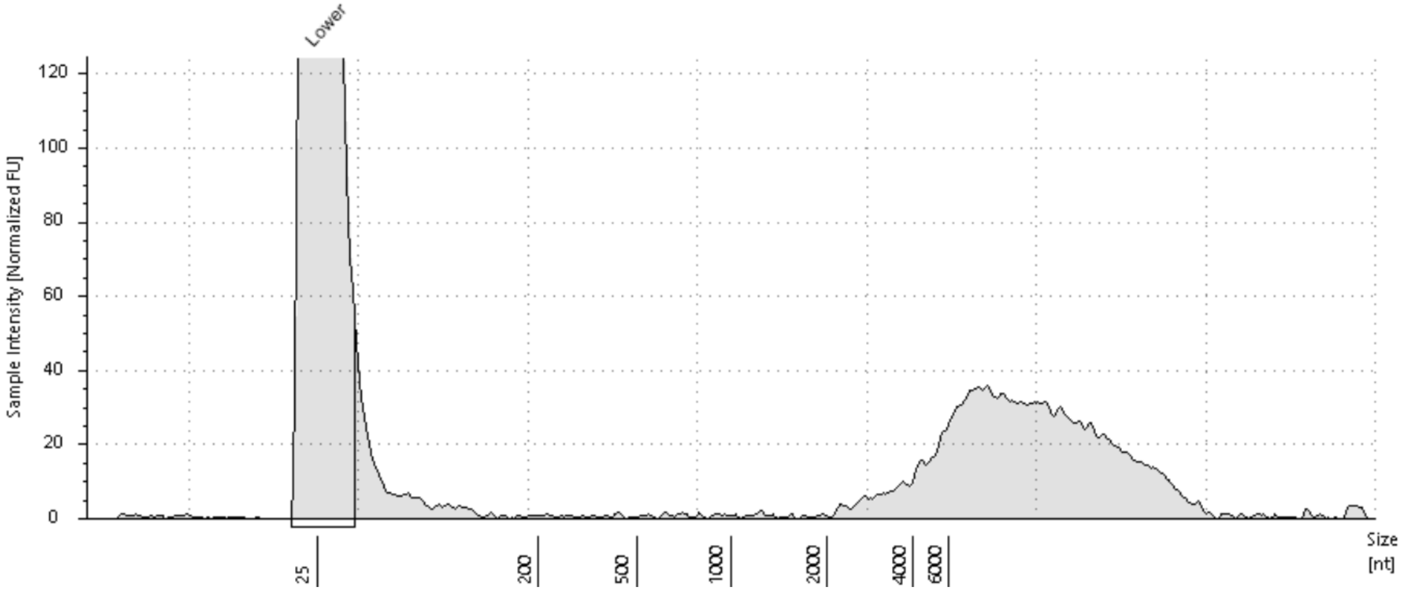

Sample Table

Well	RINᵉ	28S/18S (Area)	Conc. [pg/µl]	Sample Description	Alert	Observations
H1	-	-	87.4	22082R-05-19		

Peak Table

Size [nt]	Calibrated Conc. [pg/µl]	Assigned Conc. [pg/µl]	Peak Molarity [pmol/l]	% Integrated Area	Peak Comment	Observations
25	700	700	82400	-		Lower Marker

A2: 22082R-05-20

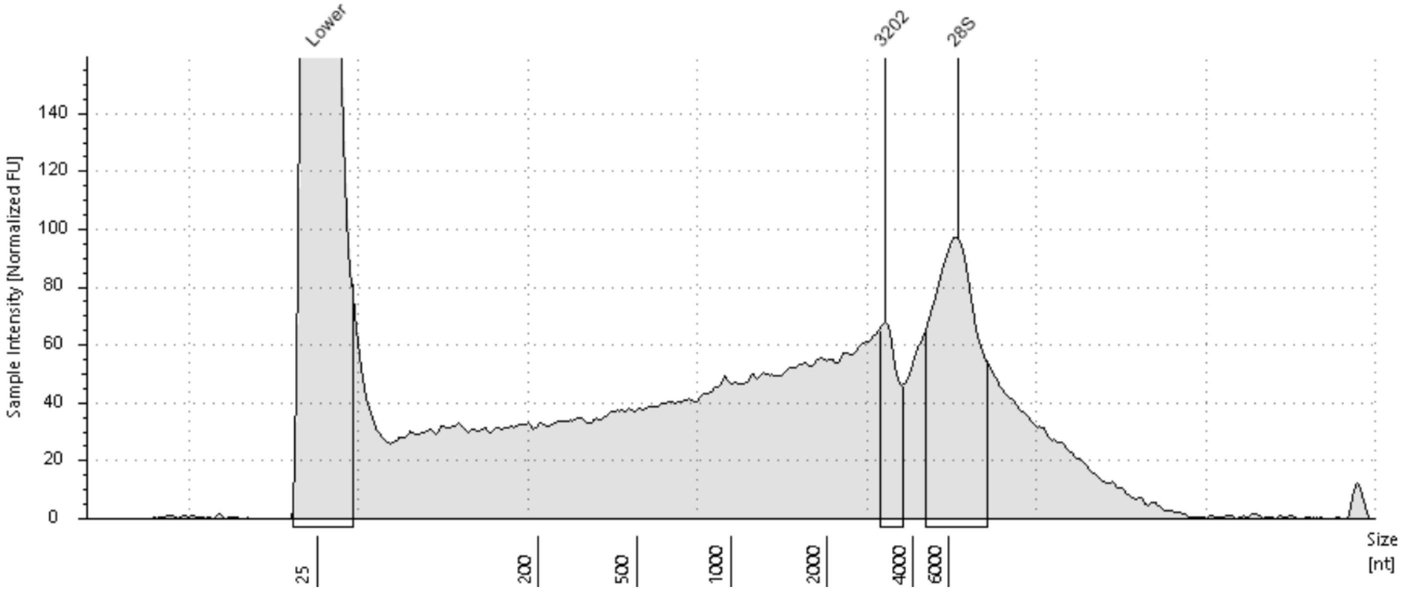

Sample Table

Well	RIN^e	28S/18S (Area)	Conc. [pg/µl]	Sample Description	Alert	Observations
A2	-	-	373	22082R-05-20		

Peak Table

Size [nt]	Calibrated Conc. [pg/µl]	Assigned Conc. [pg/µl]	Peak Molarity [pmol/l]	% Integrated Area	Peak Comment	Observations
25	700	700	82400	-		Lower Marker
3202	16.0	-	14.7	22.12		
6696	56.5	-	24.8	77.88		28S

B2: 22082R-05-21

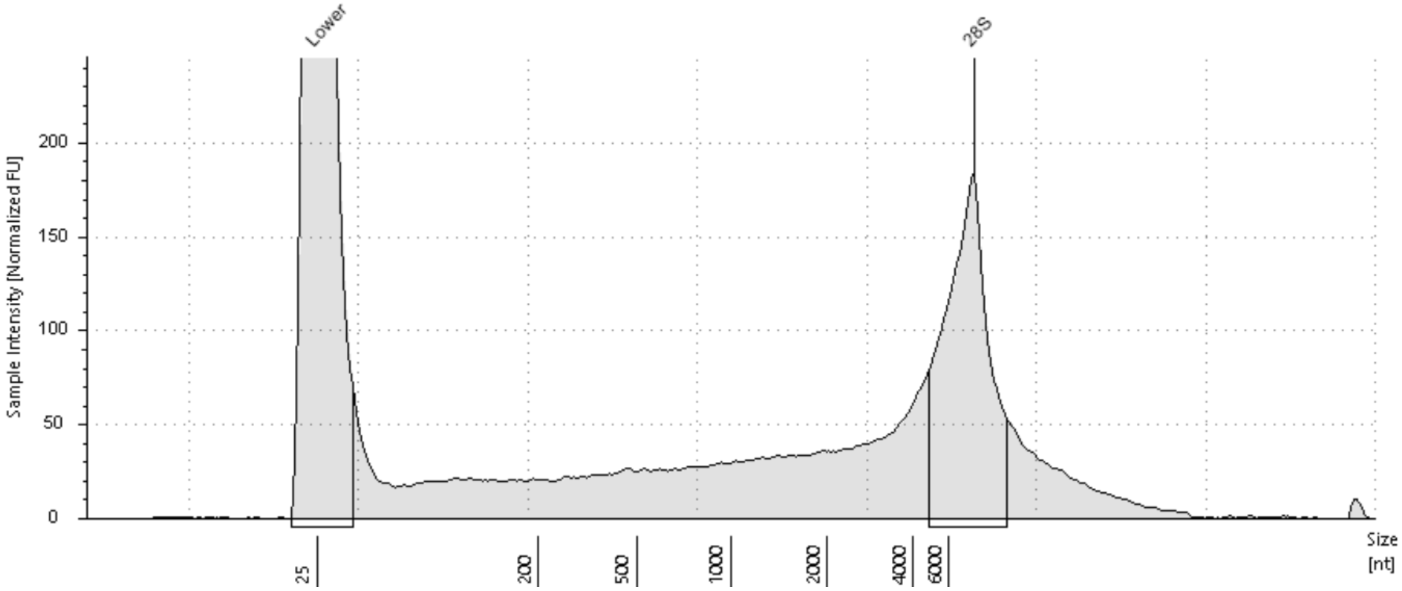

Sample Table

Well	RIN[e]	28S/18S (Area)	Conc. [pg/µl]	Sample Description	Alert	Observations
B2	-	-	321	22082R-05-21		

Peak Table

Size [nt]	Calibrated Conc. [pg/µl]	Assigned Conc. [pg/µl]	Peak Molarity [pmol/l]	% Integrated Area	Peak Comment	Observations
25	700	700	82400	-		Lower Marker
8023	101	-	37.0	100.00		28S

C2: 22082R-05-22

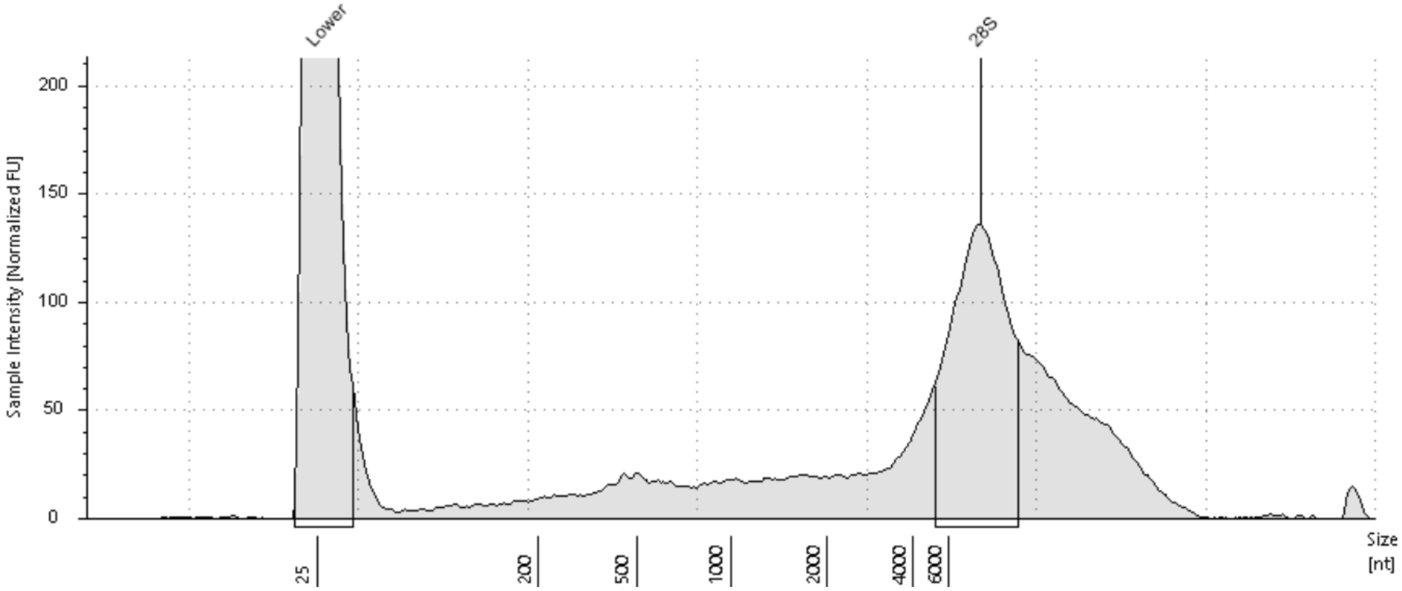

Sample Table

Well	RIN^e	28S/18S (Area)	Conc. [pg/µl]	Sample Description	Alert	Observations
C2	-	-	282	22082R-05-22		

Peak Table

Size [nt]	Calibrated Conc. [pg/µl]	Assigned Conc. [pg/µl]	Peak Molarity [pmol/l]	% Integrated Area	Peak Comment	Observations
25	700	700	82400	-		Lower Marker
8525	100	-	34.6	100.00		28S

D2: 22082R-05-23

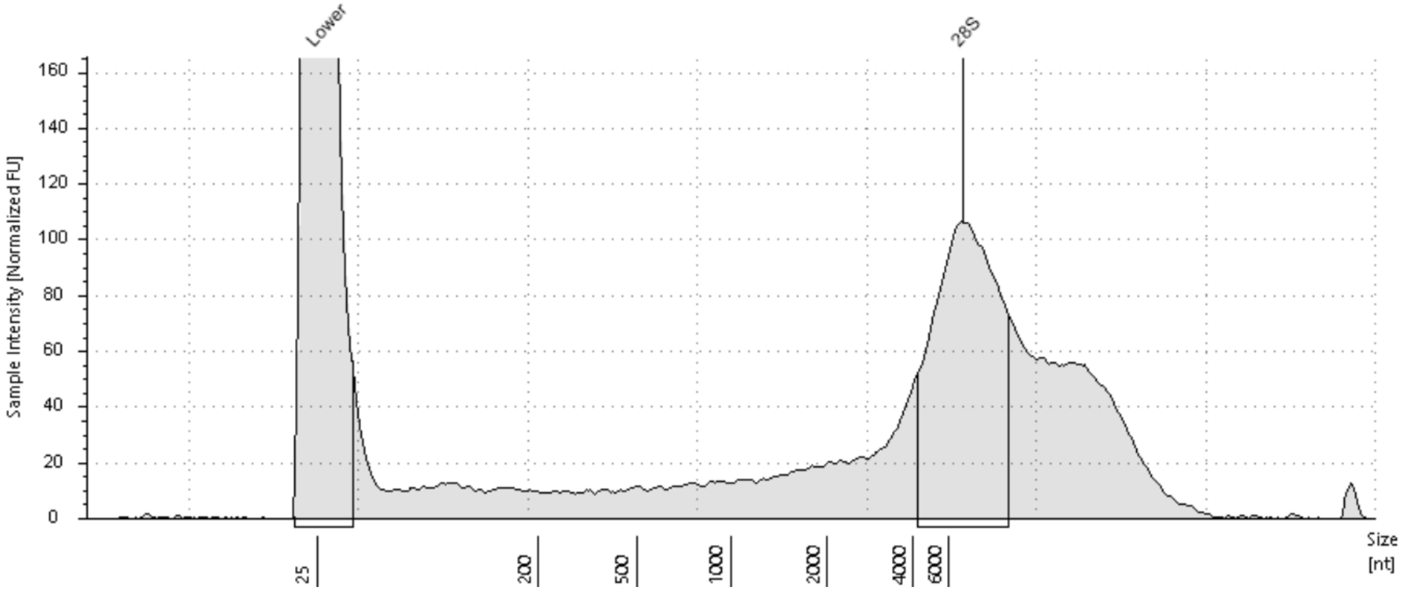

Sample Table

Well	RIN^e	28S/18S (Area)	Conc. [pg/µl]	Sample Description	Alert	Observations
D2	-	-	281	22082R-05-23		

Peak Table

Size [nt]	Calibrated Conc. [pg/µl]	Assigned Conc. [pg/µl]	Peak Molarity [pmol/l]	% Integrated Area	Peak Comment	Observations
25	700	700	82400	-		Lower Marker
7010	94.9	-	39.8	100.00		28S

E2: 22082R-05-24

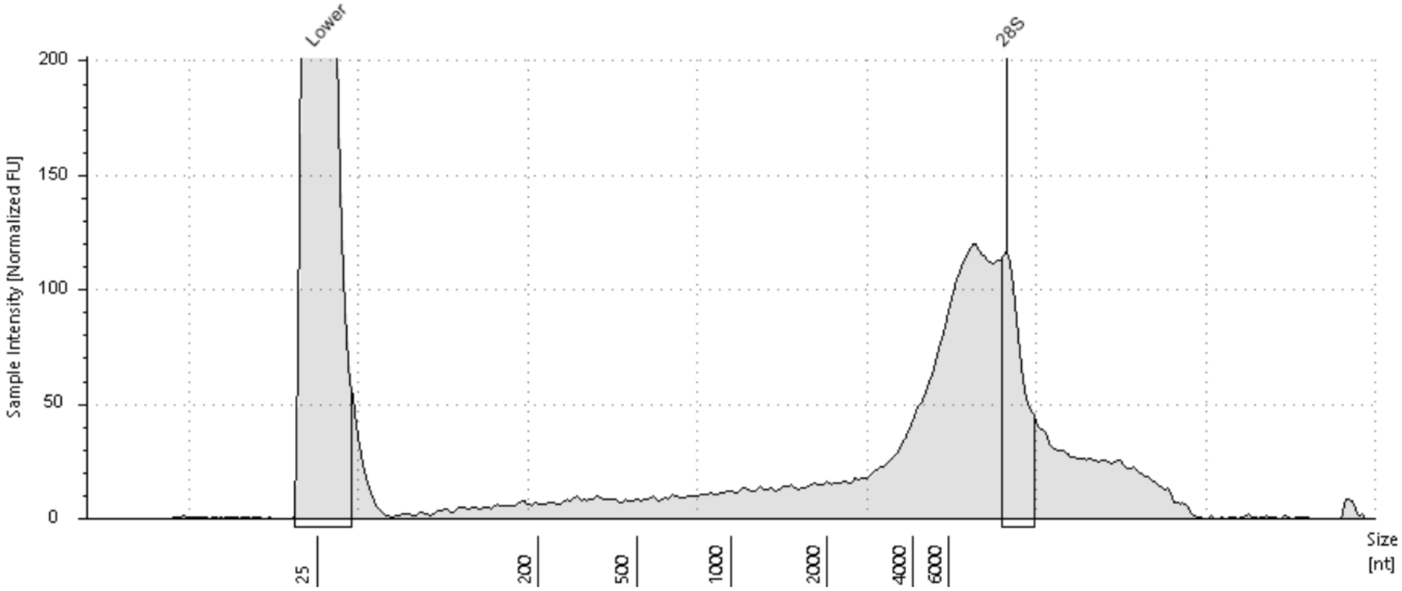

Sample Table

Well	RIN[e]	28S/18S (Area)	Conc. [pg/µl]	Sample Description	Alert	Observations
E2	-	-	238	22082R-05-24		

Peak Table

Size [nt]	Calibrated Conc. [pg/µl]	Assigned Conc. [pg/µl]	Peak Molarity [pmol/l]	% Integrated Area	Peak Comment	Observations
25	700	700	82400	-		Lower Marker
11672	34.1	-	8.59	100.00		28S
