# Supplementary material for: Identification of State-Specific Proteomic and Transcriptomic Signatures of Microglia-Derived Extracellular Vesicles
Source: Mol Cell Proteomics. 2023 Nov 11;22(12):100678. doi: 10.1016/j.mcpro.2023.100678 (PMC10755493; doi:10.1016/j.mcpro.2023.100678)
Supplement: Supplemental Figures S1–S4 [file mmc17.docx]

**SUPPLEMENTARY INFORMATION**

**Identification of state-specific proteomic and transcriptomic signatures of microglia-derived extracellular vesicles**

Juliet V. Santiago^1,2^, Aditya Natu^1,2^, Christina C. Ramelow^1,2^, Sruti Rayaprolu^1,2^, Hailian Xiao^1,2^, Vishnu Kumar^1,2^, Prateek Kumar^1,2^, Nicholas T. Seyfried^1,2,3^, Srikant Rangaraju^1,2^

*1. Department of Neurology, Emory University,201 Dowman Drive Atlanta, Georgia, 30322, United States of America*

*2. Center for Neurodegenerative Diseases, Emory University, Atlanta, GA 30322, USA*

*3. Department of Biochemistry, Emory University, Atlanta, GA 30322, USA*

**SUPPLEMENTARY FIGURES (4)**

**
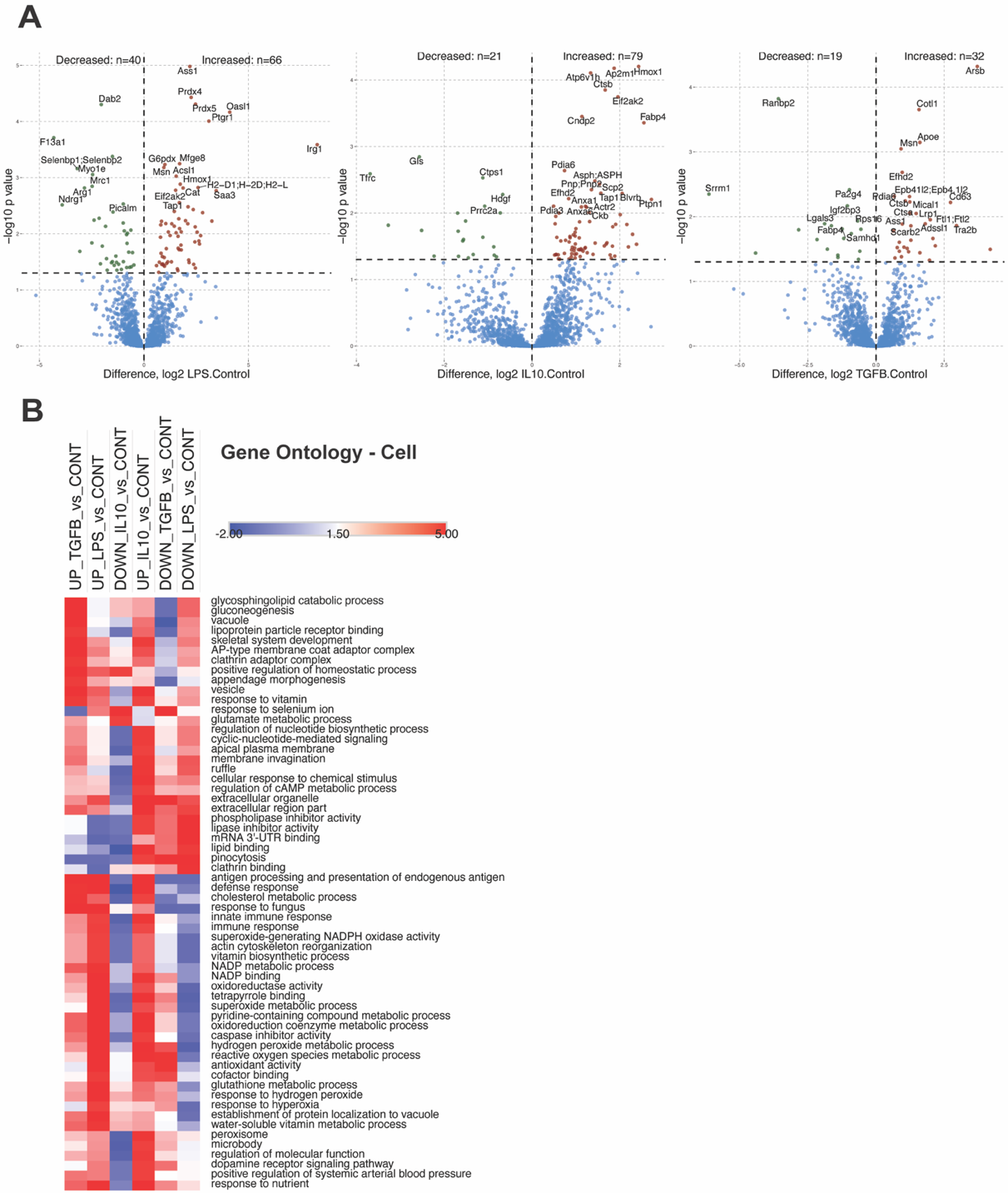
**

**Supplemental Figure 1: BV2 Cell Proteome. A.** Volcano plots showing differentially enriched proteins in EV proteome – IL10-CTL , LPS-CTL, TGF-β-CTL. **B.** Heatmap representation, based on enrichment Z-scores, of Gene Ontology for polarized cells (padj.<0.05).


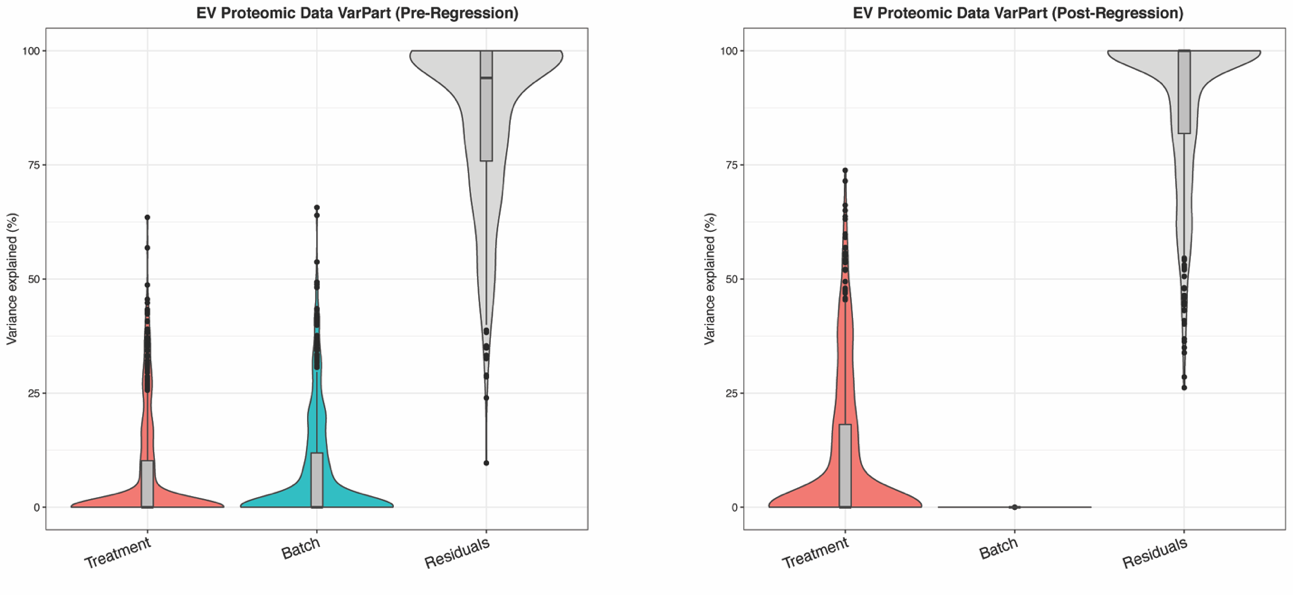


**Supplemental Figure 2: Pre and Post Regression Variance Partition Plots for BV2 EV Proteome**


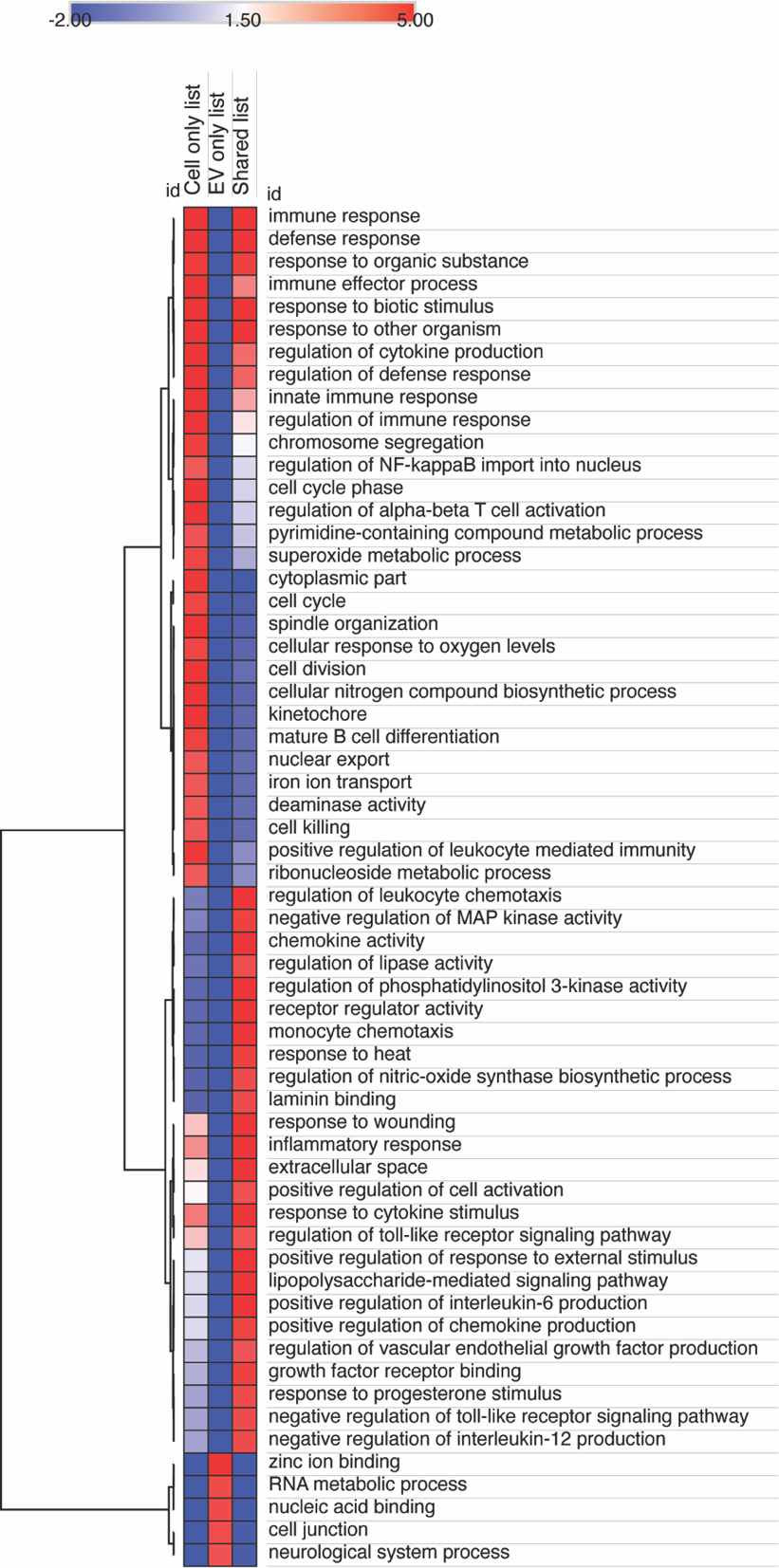


**Supplemental Figure 3:** Heatmap representation, based on enrichment Z-scores, of Gene Ontology from comparison of 2,949 upregulated mRNAs from BV2 cells treated with LPS to 1,040 upregulated mRNAs from LPS treated BV2-derived EVs (Figure 5F).


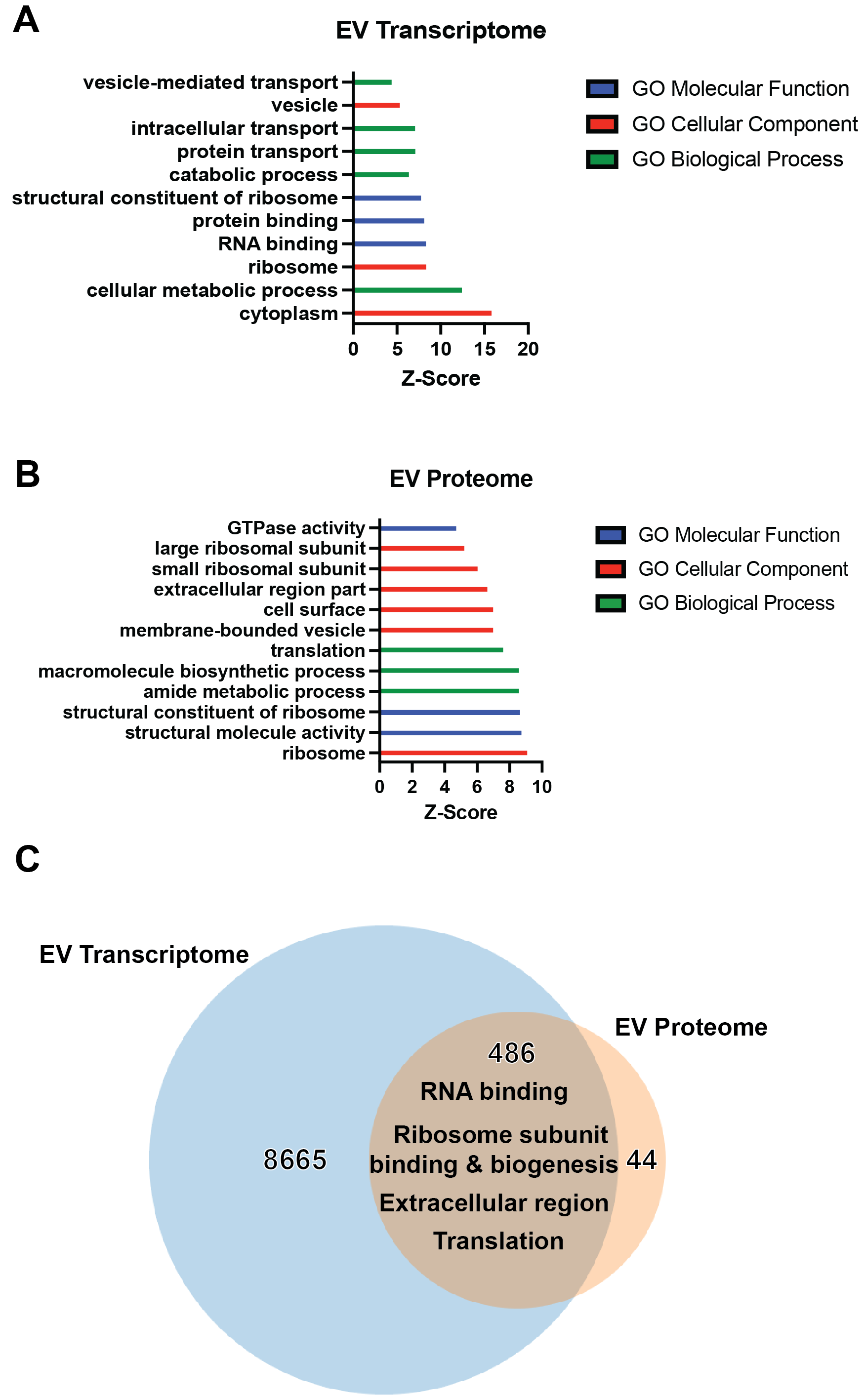


**Supplemental Figure 4:** Integrative analysis of EV proteomics and EV transcriptomics reveal common enrichment of RNA subunits, RNA binding, and translation. **A.** Top enriched pathways in the EV transcriptome. **B.** Top enriched pathways identified in the EV proteome. **C.** Venn diagram showing overlapping proteome and transcriptome pathways enriched in EVs.
