# Supplementary material for: Identification of State-Specific Proteomic and Transcriptomic Signatures of Microglia-Derived Extracellular Vesicles
Source: Mol Cell Proteomics. 2023 Nov 11;22(12):100678. doi: 10.1016/j.mcpro.2023.100678 (PMC10755493; doi:10.1016/j.mcpro.2023.100678)
Supplement: Supplemental Data [file mmc18.docx]

**Description of Supplemental data**

File name: Supplemental data 1

File format: .pdf

Title of data: Raw western blots for CD9, TSG101, and Calnexin

Description of data: These data are associated with Figure 1E.

File name: Supplemental data 2

File format: .xls

Title of data: Proteomics details for Cells and EVs datasets

Description of data: Accession #, # of distinct peptides assigned for each protein, % coverage of each protein assigned and Quantification measurements for each protein for both the Cells and EVs datasets.

File name: Supplemental data 3

File format: .xls

Title of data: Log2 transformed protein abundances, differential enrichment analyses, and Gene set enrichment analysis from BV2 cell proteome.

Description of data: These data are associated with Figures 2 and 3. P values are from T-test (2 tailed, assuming equal variance). Benjamin-Hochberg false-discovery-rate (BH-FDR) p values are also shown.

File name: Supplemental data 4

File format: .xls

Title of data: Log2 transformed protein abundances, differential enrichment analyses, and gene set enrichment analysis from BV2-derived EV proteome.

Description of data: These data are associated with Figures 2, 3, and 4. P values are from T-test (2 tailed, assuming equal variance). Benjamin-Hochberg false-discovery-rate (BH-FDR) p values are also shown.

File name: Supplemental data 5

File format: .xls

Title of data: Comparison of BV2 cell proteome to BV2-dervied EV proteome

Description of data: Log2 transformed protein abundances, differential enrichment analyses, and gene set enrichment analysis from BV2 cell versus EV proteome. These data are associated with Figure 2. P values are from T-test (2 tailed, assuming equal variance). Benjamin-Hochberg false-discovery-rate (BH-FDR) p values are also shown.

File name: Supplemental data 6

File format: .xls

Title of data: Gene set enrichment analysis of proteins in BV2 EV proteome vs. Non-microglial EV proteome

Description of data: List of proteins and gene set enrichment analysis corresponding to Venn diagrams in Figure 2C-F.

File name: Supplemental data 7

File format: .pdf

Title of data: RNA Quality Report from Admera of BV2 cells and extracellular vesicles

Description of data: RNA Quality Report from Admera of BV2 cells and extracellular vesicles (EVs)

File name: Supplemental data 8

File format: .xls

Title of data: Raw counts, post normalized counts, differential expression analyses, and gene set enrichment analyses from BV2 cell vs EV mRNA sequencing dataset.

Description of data: These data are associated with Figure 5. P values are from T-test (2 tailed, assuming equal variance). Benjamin-Hochberg false-discovery-rate (BH-FDR) p values are also shown.

File name: Supplemental data 9

File format: .xls

Title of data: Raw counts, post normalized counts, differential expression analyses, and gene set enrichment analyses from BV2 cell mRNA sequencing dataset.

Description of data: These data are associated with Figure 5. P values are from T-test (2 tailed, assuming equal variance). Benjamin-Hochberg false-discovery-rate (BH-FDR) p values are also shown.

File name: Supplemental data 10

File format: .xls

Title of data: Raw counts, post normalized counts, differential expression analyses, and gene set enrichment analyses from BV2-derived EV mRNA sequencing dataset.

Description of data: These data are associated with Figure 5. P values are from T-test (2 tailed, assuming equal variance). Benjamin-Hochberg false-discovery-rate (BH-FDR) p values are also shown.

File name: Supplemental data 11

File format: .xls

Title of data: Integrative analysis of EV proteomics and EV transcriptomics

Description of data: These data are associated with Supplemental Figure 4. List of all proteins in EVs and all mRNAs in EVs following normalization and 50% missingness threshold. Gene set enrichment analysis of all proteins in EVs, all mRNAs in EVs, and corresponding reference lists.

File name: Supplemental data 12

File format: .xls

Title of data: Raw counts, post normalized counts and differential expression analyses, from BV2 Cell miRNA sequencing dataset.

Description of data: These data are associated with Figure 6. P values are from T-test (2 tailed, assuming equal variance). Benjamin-Hochberg false-discovery-rate (BH-FDR) p values are also shown.

File name: Supplemental data 13

File format: .xls

Title of data: Raw counts, post normalized counts, differential expression analyses, and gene set enrichment analyses from BV2 EV miRNA sequencing dataset.

Description of data: These data are associated with Figure 6. P values are from T-test (2 tailed, assuming equal variance). Benjamin-Hochberg false-discovery-rate (BH-FDR) p values are also shown.

File name: Supplemental data 14

File format: .xls

Title of data: BV2-dervied EV other small RNA sequencing datasets (circRNA, piRNA, snoRNA, snRNA, tRNA).

Description of data: Raw counts from BV2-dervied EV other small RNA sequencing datasets (circRNA, piRNA, snoRNA, snRNA, tRNA).

File name: Supplemental data 15

File format: .xls

Title of data: Raw counts, post normalized counts, and differential expression analyses from BV2 Cell vs EV miRNA sequencing dataset.

Description of data: These data are associated with Figure 6. P values are from T-test (2 tailed, assuming equal variance). Benjamin-Hochberg false-discovery-rate (BH-FDR) p values are also shown.

File name: Supplemental data 16

File format: .xls

Title of data: Post normalized counts, differential expression analyses, gene set enrichment analyses, and FET analysis from BV2 Responder Cells bulk RNA sequencing dataset.

Description of data: These data are associated with Figure 7. P values are from T-test (2 tailed, assuming equal variance). Benjamin-Hochberg false-discovery-rate (BH-FDR) p values are also shown.
